# A Review on Biodegradable Materials of Sustainable Soft Robotics and Electronics

**DOI:** 10.1002/advs.202510320

**Published:** 2025-08-21

**Authors:** Yizhu Xie, Jiaxin Han, Xingyi Dai, Xuyang Zhang, Kangwen Xiao, Junhui Huang, Yajun Cao, Aihua Zhong, Long‐Biao Huang

**Affiliations:** ^1^ Key Laboratory of Optoelectronic Devices and Systems of Ministry of Education and Guangdong Province College of Physics and Optoelectronic Engineering Shenzhen University Shenzhen 518060 P. R. China; ^2^ National Key Laboratory of Green and Long‐Life Road Engineering in Extreme Environment Shenzhen University Shenzhen 518060 P. R. China

**Keywords:** biodegradable materials, degradation mechanism, sensors, sustainable soft robots

## Abstract

With the increasing concerns over environmental pollution and healthcare demands, biodegradable materials are showing promising applications in the field of soft robotics. As two fundamental components of soft robots, actuators and soft sensors predominantly rely on non‐biodegradable materials for fabrication, which raises significant environmental concerns. This review provides a comprehensive summary of current advancements in the utilization of biodegradable materials for soft robotics sensors. Biodegradable materials mainly include degradable metals, biodegradable polymers (such as cellulose and chitosan). Due to their environmental friendliness and biodegradability, these materials have shown competitive potential as excellent alternatives to traditional non‐biodegradable sensor materials. Sensors for soft robotics based on biodegradable materials, including tactile sensors, strain/pressure sensors, temperature sensors, humidity sensors, olfactory sensors, and implantable sensors, are systematically summarized. Although biodegradable sensors show great potential in sustainable soft robots, they still face challenges such as degradation rate control, insufficient mechanical strength, and large‐scale production. Future research should focus on the integration of multifunctional materials, precise regulation of degradation mechanisms, and compatibility with traditional electronic components. This review aims to provide a comprehensive understanding of the development of biodegradable sensors, promote their widespread application in green robotics technologies, and contribute to the realization of global sustainable development goals.

## Graphical abstract

Biodegradable materials are gaining increasing attention in soft robotics and electronics due to their environmental friendliness, showing great potential for sustainability. In this review, the classification of biodegradable materials, their applications in the field of soft robotics and electronics, as well as the challenges and future prospects, are summarized and discussed.

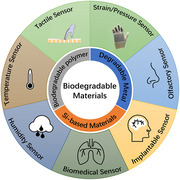

## 1 Introduction

Traditional robots rely on metals and hard plastics, and are adept at high‐precision repetitive tasks, but their performance is limited in complex, unstructured environments such as narrow pipelines and human interiors. Last decades, the rise of soft robotics has filled this gap, as its biomimetic design and flexible materials endow it with excellent deformability, safety, and environmental adaptability.^[^ ^1^, ^2^ ^]^ Therefore, developing soft robots is not only a requirement for technological upgrading, but also an inevitable choice to expand the application boundaries of robots.^[^ ^3^ ^]^ Its unique flexibility and adaptability give it extensive application potential in diverse fields such as healthcare,^[^ ^4^, ^5^ ^]^ rescue operations,^[^ ^6^ ^]^ industry,^[^ ^7^ ^]^ and exploration.^[^ ^8^ ^]^

In the future, soft robots will increasingly enter our daily lives, which will lead to raise environmental issues at the end of their life. Along with environmental problems and resource shortages intensify, the application of sustainable materials in the field of soft robotics needs to be explored urgently. Consequently, the utilization of sustainable materials is not merely a requirement for technological development but also an inevitable choice in response to the global ecological crisis. The feasible solutions include: First, utilization of recyclable materials, such as thermoplastic elastomers, which can be remelted and reshaped for multiple cycles of reuse; Second, incorporation of renewable or natural resources, like natural fiber and mycelium, among other bio‐based materials, which have short growth cycles and low carbon footprints, and can replace some synthetic materials; Third, optimizing manufacturing processes by adopting technologies such as 3D printing and low‐temperature forming to reduce energy consumption;^[^ ^9^ ^]^ And fourth, developing self‐healing materials, which extending the robot's lifespan and indirectly reducing material replacement and waste.^[^ ^10^, ^11^ ^]^ Moreover, another significant approach involves the utilization of biodegradable materials, which can decompose into harmless substances under specific conditions.^[^ ^10^, ^12^, ^13^, ^14^ ^]^

To fully realize their potential, soft robots must achieve seamless integration of actuator, sensors, and controller.^[^ ^14^, ^15^ ^]^ Actuators and soft sensors are the two fundamental components of soft robots. Their relationship is akin to the muscles and nervous system in biological organisms, enabling intelligent perception and action execution through dynamic interaction. Specifically, on the one hand, actuators primarily serve to drive the deformation of flexible structures, transmit mechanical forces, and adapt to complex environments.^[^ ^16^, ^17^ ^]^ On the other hand, sensors help robots perceive and adapt to their environment, and support efficient and safe task execution in complex scenarios, making them a critical component for achieving high precision, flexibility, and autonomy in soft robots.^[^ ^18^ ^]^ However, most of the actuators and sensors have been fabricated by non‐biodegradable synthetic materials (such as silicone and petroleum‐based polymers), which gradually expose environmental issues such as the accumulation of electronic waste and ecological pollution. Biodegradable materials have become a key breakthrough in addressing the aforementioned contradictions. In recent years, many researchers have been dedicated to develop biodegradable soft robots,^[^ ^19^, ^20^, ^21^, ^22^ ^]^ in which all the components, encompassing actuators and soft sensors, are engineered to be biodegradable. Despite significant advancements, several critical challenges remain to be overcome to fabricate fully biodegradable soft robots. For example, sensors are composed of several components, such as sensing units, power supplies, and encasing structures, and different components may utilize one or more different materials, such as metal, semiconductors, and insulators. Fabricating all these components from biodegradable materials poses a significant challenge. At the same time, the design of biodegradable sensors requires balancing environmental compatibility and functional stability. The degradation rate of the materials must align with the expected lifespan of the robot. For example, sensors used for short‐term environmental monitoring should degrade quickly after completing their tasks, while implantable medical devices should operate stably in the body for several weeks before degrading.^[^ ^23^ ^]^

Recently, several reviews have extensively explored the application of sustainable materials in sensors and soft robotics.^[^ ^10^, ^12^, ^13^ ^]^ Therefore, this article focuses on biodegradable materials for the sensors of soft robotic systems. We first examine different biodegradable materials for soft sensors, including biodegradable metals, polymers, and silicon‐based materials. Subsequently, we outline fundamental sensing principles and systematically classify common sensors in soft robotics, such as strain/pressure sensors, tactile sensors, environmental monitors, and biodegradable implantable sensors. Challenges and outlooks of biodegradable materials in the sensors of soft robotics are given at the end.

## 2 Materials

Biodegradable materials could significantly mitigate the environmental impact at the end of a robot's life. For the realization of fully sustainable soft robotics, all components, including the actuator and sensors, should be fabricated from biodegradable materials. Nevertheless, soft robots engineered for diverse applications exhibit divergent requirements for biodegradability. This imposes specific requirements on material selection.

### 2.1 Requirements for Biodegradable Materials of Soft Robotics

In the realm of sustainable soft robotics, the full degradation of all components of sensors is a critical requirement. Nevertheless, most actuators and soft sensors in soft robotics often do not exclusively comprise degradable components, or the degradation rates of their components exhibit significant disparities. For medical soft robots utilized in applications such as wound treatment or drug delivery, degradation must occur under physiological conditions to render them truly bioresorbable devices. Therefore, the lifespan of all materials should be limited to timescales comparable to human tissue healing or regeneration processes, and each degradation product must be non‐cytotoxic. Such medical robots require complete metabolism of all constituents.

However, even if a material is classified as biodegradable, degradation may not ensue at all when it is immersed in an unsuitable environment. Hence, materials should be carefully selected based on the intended application of the soft robot. For instance, Ecoflex, an elastomer utilized in soft robotics, undergoes complete decomposition within ≈80 days under industrial composting conditions.^[^ ^12^ ^]^ Conversely, when immersed in seawater, its degradation period is significantly protracted. In contrast, search and rescue robots, on the other hand, are engineered to degrade in organic waste and compost environments, whereas marine robots designed for underwater operations require materials that disintegrating in seawater. The lifetime of soft robotics relies on the chemical stability of the materials, which is determined by its degradation rate of different materials. Therefore, both material selection and device design demand meticulous consideration.

### 2.2 Degradable Materials

To achieve sustainable soft robotics, a variety of degradable materials have been proposed to make the components of robotics, including substrate, interconnects, semiconductor components, and encapsulation. These biodegradable materials include metals (such as magnesium (Mg),^[^ ^24^, ^25^ ^]^ zinc (Zn),^[^ ^26^ ^]^ iron (Fe),^[^ ^27^ ^]^ molybdenum (Mo),^[^ ^28^ ^]^ silver (Ag)^[^ ^29^, ^30^ ^]^), polymers (such as polyvinyl alcohol (PVA),^[^ ^31^ ^]^ Ecoflex,^[^ ^32^ ^]^ cellulose,^[^ ^33^, ^34^ ^]^ silicon‐based materials (such as Si (silicon), SiO_2_(silicon oxide), Si_3_N_4_(silicon nitride))^[^ ^35^, ^36^ ^]^ and their composites, as summarized in **Table** **1**. In the following sections, we will elaborate on the properties of various materials that can be used for fabricating biodegradable sensors.

**Table 1:** Summary of degradable materials used in sensors and their advantages/disadvantages.

Material	Sensor Component	Advantages	Disadvantage	Refs.
**Degradable Metals**				
Magnesium (Mg)	Interconnects, electrodes	Easy processing, biocompatible	Low mechanical strength, low fatigue resistance, fast dissolution kinetics	[24, 25]
Zinc (Zn)	Interconnects, electrodes	Easy processing, modest dissolution kinetics, and biocompatible	Low mechanical strength, low fatigue resistance, and slow degradation	[41]
Iron (Fe)	Interconnects, electrodes	Good mechanical properties, homogeneous dissolution, and biocompatible	Slow degradation	[27]
Tungsten (W)	Interconnects, electrodes	High strength and Young's modulus	Very slow degradation	[28]
Molybdenum (Mo)	Interconnects, electrodes	High strength and Young's modulus, compatible with magnetic resonance, biocompatible	Very slow degradation	[28]
Silver (Ag)	Interconnects, electrodes	Easy processing, stable chemical properties, and biocompatible	Very slow degradation	[43]
**Polymers**				
Ecoflex	Support, substrate, adhesive, encapsulation	High modulus of elasticity, biocompatible	Slow degradation, temperature sensitive.	[49]
Gelatin	Support, substrate, adhesive, encapsulation	Low gelation temperature, non‐toxic, high water absorption, biocompatible	Weak mechanical properties	[52, 53, 54]
Chitosan	Support, substrate, adhesive, encapsulation	Enzymatically degraded by lysozyme and chitosanase enzymes	Water‐insoluble, unstable	[57, 58]
Silk fibroin (SF)	Support, substrate, adhesive, encapsulation	Easy processing, biocompatible, transparent, and good mechanical properties		[70, 71, 72]
Polylactide (PLA)	Packaging, wires, optical fiber	Degraded by the hydrolysis of ester bonds without requiring any enzymes	Slow degradation rate, hydrophobicity, and low impact toughness	[76]
Poly(L‐lactide) (PLLA)	Transducer	Low temperature processing, oxide‐free interfaces, good electronic and ionic conductivity	Modest piezoelectric response	[78]
Polyvinyl alcohol (PVA)	Substrate, adhesive layer	Easy processing, good water solubility, and biocompatible	Relatively low mechanical strength	[31]
Polycaprolactone (PCL)	Substrate, bonding, and sealing	Semi‐rigid at room temperature	Slow degradation rate, low stiffness	[87]
Polypyrrole (PPy)	Electrodes	High conductivity, good electrochemical stability, and good mechanical properties	Complex doping process	[96, 97, 98]
Poly(3,4‐ethylenedioxythiophene)(PEDOT)	Electrodes			[102]
Polyaniline (PANI)	Electrodes	Good electrochemical stability		[110]
**Silicon‐based Materials**				
Silicon (Si)	Functional layer, substrate	Compatible with established microfabrication techniques, piezoresistive	Rigid and stiff material	[35, 36]
Silicon oxide (SiO_2_)	Encapsulation layer, dielectric layer of the sensor	Compatible with established microfabrication techniques		[113]
Silicon nitride (Si_3_N_4_)	Dielectric layer of the sensor	Compatible with established microfabrication techniques		[113]

#### 2.2.1 Degradable Metals

Degradable metals represent a distinct category of metallic materials characterized by their ability to completely decompose into biocompatible constituents under specific environmental conditions, such as biofluids or activation via trigger mechanisms. In the field of degradable sensors, commonly used metals include magnesium (Mg), iron (Fe), zinc (Zn), tungsten (W), molybdenum (Mo), manganese (Mn), and silver (Ag). Despite the limited contribution of enzymes to metal degradation, where hydrolysis represents the predominant reaction mechanism, degradation kinetics are primarily governed regulated by temperature, pH, and salt content in the solution. Metals and their alloys serve as critical components of many sensors, and a comprehensive understanding of their properties is essential for the effective development of sensor technologies. Yin et al.^[^ ^27^ ^]^ have studied the electrical degradation rates and associated changes in the microstructure of five of these metals in deionized (DI) water.

Among these, Mg stands out as the preferred material for implantable devices due to its high conductivity, ease of processing, and rapid degradation kinetics.^[^ ^24^, ^25^ ^]^ Mg is used for making substrates, connecting wires, and can also be applied in wireless signal transmission systems.^[^ ^37^ ^]^ Pressure sensors^[^ ^24^ ^]^ and temperature sensors^[^ ^38^ ^]^ made from Mg for implantable devices have also been reported. In water or biological fluids, Mg degrades at a rate of several micrometers per day. Degradation mechanisms of Mg in DI water or biofluids can be presented as follows:^[^ ^39^ ^]^

(1) $$\mathrm{Mg}+2H_{2}O\to \mathrm{Mg}{\left(\mathrm{OH}\right)}_{2}↓+H_{2}↑$$

However, the relatively fast degradation rate of Mg and its alloys necessitates the use of encapsulation layers to modulate the degradation rate in some cases.^[^ ^40^ ^]^

Zinc (Zn) shares a similar degradation mechanism with Mg but exhibits a slower degradation rate in solution, making it unsuitable for short‐term devices. Experimental results indicate that the electrochemical dissolution rate of Zn in Hank's solution at room temperature is 0.3 µm h^−1^, much slower than the degradation rate of Mg (4.8 µm h^−1^).^[^ ^41^ ^]^ The degradation rate of Zn can be controlled by its structure or encapsulation.^[^ ^26^ ^]^ Degradation mechanisms of Zn in water can be presented as follows:

(2) $$\mathrm{Zn}+2H_{2}O\to \mathrm{Zn}{\left(\mathrm{OH}\right)}_{2}↓+H_{2}↑$$

The surface of Zn does not degrade uniformly, ZnO and Zn(OH)_2_ are the dominant surface products. It is estimated that Zn oxide dissolves at 120–170 per day.^[^ ^42^ ^]^

In Hank's solution at room temperature, the electrochemical dissolution rate of Fe is 7–9 µm h^−1^.^[^ ^27^ ^]^ Its degradation reaction involves the oxidation of ferrous ions (Fe^2^⁺) in an oxygen‐rich environment, ultimately forming slowly dissolving hydroxides. Zn and Fe were used to fabricate different biodegradable sensors, for example, wireless RF pressure sensor^[^ ^42^ ^]^ and a soil moisture sensor.^[^ ^26^ ^]^

Molybdenum (Mo) and tungsten (W) demonstrate relatively slow dissolution rates, 0.2–0.6 and 0.2–0.8 nm h^−1^, respectively, in Hank's solution (pH 5–8). Both of them could be used to make electrodes in biodegradable sensors.^[^ ^28^ ^]^

Silver (Ag) can also be used to fabricate biodegradable sensors, such as temperature or pressure sensors. It is achieved by employing innovative methods, typically in the form of nanowires or nanoparticles combined with biodegradable adhesives (e.g., PVA) to create water‐soluble conductive inks.^[^ ^29^, ^30^ ^]^ Some alloys have better mechanical and electrical properties compared to their pure metal. Since metals and metal alloys will be a key material in most sensors, so their properties need to be understood for effective development of a biodegradable sensor.^[^ ^43^ ^]^ A highly sensitive biodegradable pressure sensor was reported by using Fe–Zn bilayer electrodes and PLGA‐PCL composite membrane as an elastomeric dielectric.^[^ ^44^ ^]^

The application of degradable metals in the field of intelligent sensing can significantly enhance environmental friendliness. Karami–Mosammam et al.^[^ ^45^ ^]^ prepared a kind of wearable system based on a biodegradable battery and biomedical sensor patches, using a combination of molybdenum metal foils, a molybdenum trioxide (MoO_3_) paste, and Mg metal foil as electrode materials. The battery was capable of powering biomedical sensor patches on the skin, enabling monitoring of sodium concentration in sweat. Immersion in aqueous solution induced degradation of the battery cell. The biodegradable and metal batteries were almost completely decomposed in phosphate buffer solution after 13 weeks, as shown in Figure 2a.

#### 2.2.2 Biodegradable Polymer

**Figure 1:**
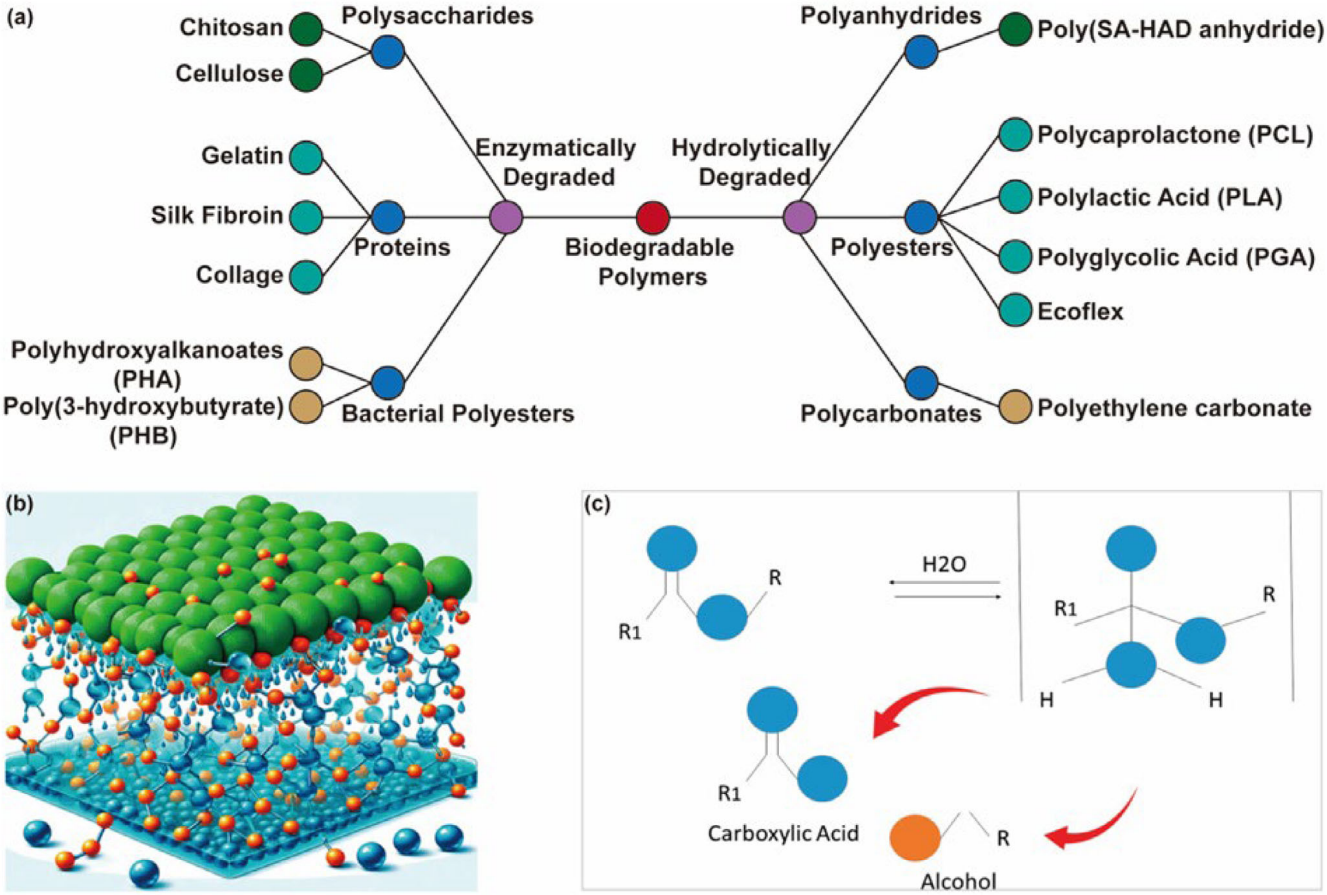
a) Classification of biodegradable polymers. b) Degradation of polymer matrix response to the presence of water; blue molecules are unbonded, orange molecules are loosely bonded, and green molecules are strongly bonded. Reproduced with permission.^[^ ^47^ ^]^ Copyright 2024, MDPI; c) The hydrolysis reaction of water with susceptible bonds of a compound forms two or more products. Reproduced with permission.^[^ ^47^ ^]^ Copyright 2024, MDPI.

Degradable polymers represent a crucial class of materials that decompose in natural environments through enzymatic or hydrolytic processes without imposing ecological harm during the process (**Figure** **1**a).^[^ ^12^ ^]^ These materials are generally easy to process, featuring tunable mechanical properties and degradation rates that could be systematically tuned through compositional adjustments. In the field of soft robotics, certain degradable polymers can be used to fabricate soft robots with on‐demand and controllable degradation. This approach effectively addresses the challenge of enabling robots to autonomously degrade upon completion of their tasks. The degradation rate of materials of soft robots must precisely match the task cycle, too fast would cause premature robot failure, while too slow would negate the environmental benefits. Most of the biodegradable soft robots are fabricated using passively degradable materials. Since these soft robots immediately initiate degradation through continuous environmental exposure, the selection and thickness determination of passively degradable materials can only be optimized based on the anticipated usage environment and material degradation rates. However, even with such considerations, controllability remains significantly limited. For achieving life reconfigurable soft robots, developing trigger‐transient materials that can both actuate and degrade on‐demand is crucial. Oh et al. proposed transient and magnetically actuating materials that can decompose under ultraviolet light and heat.^[^ ^46^ ^]^ This material was achieved by adding photoacid generator and magnetic particles (Sr‐ferrite) to poly(propylene carbonate). UV light and heat exposure could cause this material to decompose within the desired time after the final operation. Complete decomposition of the material occurred after 15 min of 365 nm UV exposure and the following 150 °C for 15 min heat treatment. Soft actuators and sensors made by trigger transient materials, which can degrade on demand, are emerging as promising alternatives for achieving efficient lifetime configuration of soft robots.

The degradation of most degradable polymers is achieved through hydrolysis reactions. The polymer degradation process in water and the corresponding hydrolysis reactions are illustrated in Figure 1b and Figure 1c.^[^ ^47^ ^]^ Many non‐conductive polymers are primarily used as substrates, encapsulation materials, adhesives, or as dielectric layers in sensors. Biodegradable conductive polymers such as polypyrrole (PPy) and polyaniline (PANI) have shown great potential across various application fields due to their unique physicochemical characteristics. These polymers not only exhibit excellent conductivity but also facilitate the flexible modulation of electrical conductivity via doping/dedoping processes, with a wide and easily adjustable conductivity range. Such characteristics render them ideal for sensors. Synthesis methods, including chemical oxidative polymerization and electrochemical polymerization, afford precise control over their morphology and performance.

Ecoflex, a high‐performance biodegradable material,^[^ ^48^ ^]^ had been widely employed in various flexible electronic devices and soft robot systems.^[^ ^49^ ^]^ It is synthesized from 1,4‐butanediol, adipic acid, and terephthalic acid. Ecoflex is typically formed by mixing a two‐component system, followed by curing at room temperature. The cured Ecoflex is semi‐transparent and exhibits excellent mechanical properties, offers high elasticity with a strain range of 600%–900% and a tensile strength of ≈50–100 kPa.^[^ ^50^ ^]^ As lacking intrinsic conductivity, Ecoflex is predominantly utilized as a support, substrate, adhesive, or encapsulation material in flexible electronics and soft robots, in which serve as a structural matrix. Under industrially composted, Ecoflex degrades completely into carbon dioxide, water, and biomass without leaving harmful residues. However, its degradation byproducts may persist in biological systems rather than being fully absorbed. And, degradation occurs very slowly in natural environments or seawater, demonstrating strong resistance to breakdown.

**Figure 2:**
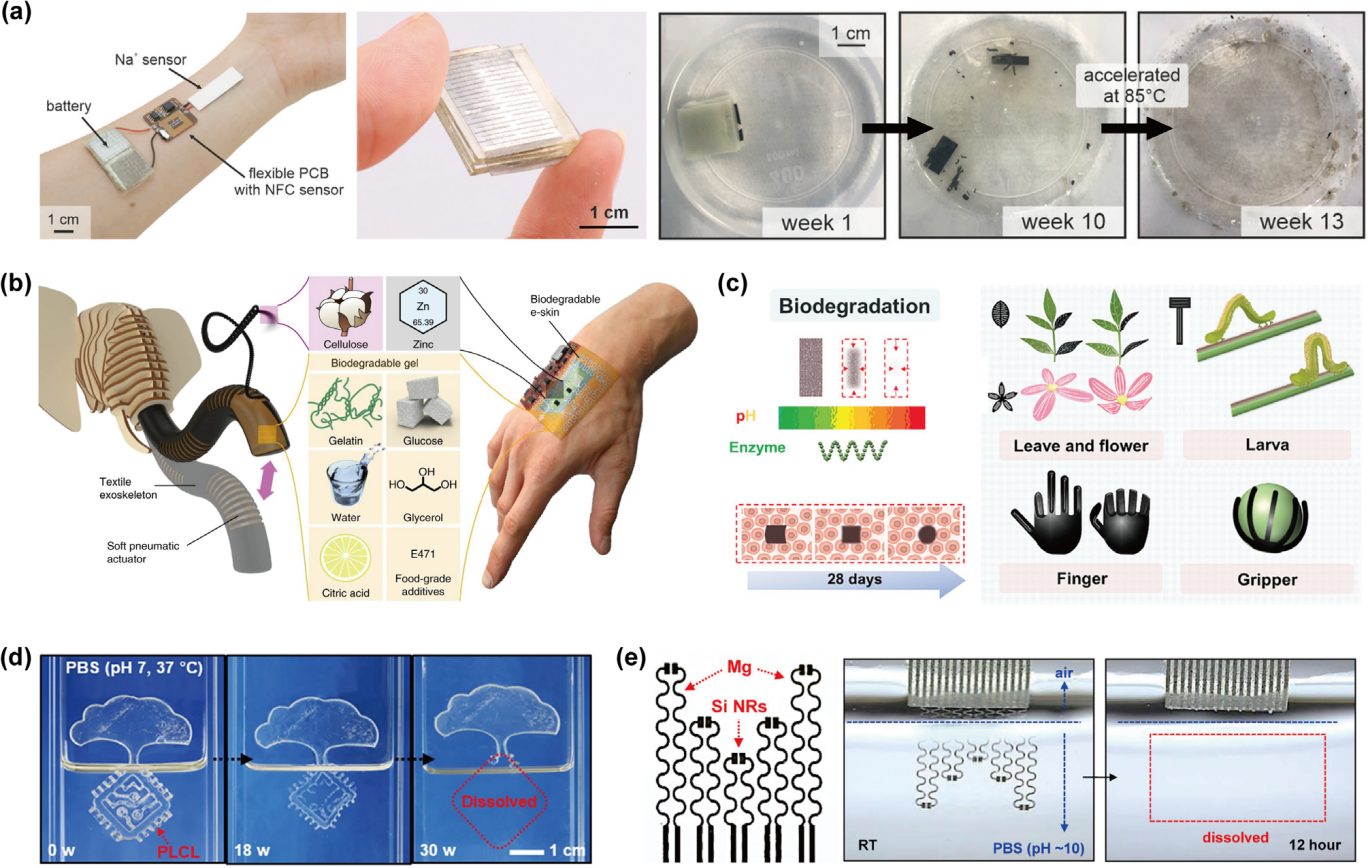
Various biodegradable materials for devices and their decomposition process. a) Sensor patch, battery based on molybdenum and Mg metal foils, physical image, and the degradation process of the biodegradable battery in phosphate buffer solution (pH 7.4). Reproduced with permission.^[^ ^45^ ^]^ Copyright 2022, John Wiley & Sons. b) Degradable gelatin‐based biogel for flexible pneumatic actuators and multifunctional electronic skin. Reproduced with permission.^[^ ^55^ ^]^ Copyright 2020, Springer Nature. c) Morphing and locomotion of the biodegradable and multifunctional chitosan‐MNP soft actuator. Reproduced with permission.^[^ ^61^ ^]^ Copyright 2024, John Wiley & Sons. d) The degradation process of PLCL film for a transient robotic gripper in phosphate‐buffered solution. Reproduced with permission.^[^ ^91^ ^]^ Copyright 2023, Springer Nature. e) A biodegradable, stretchable pH sensor based on Si‐doped nanoribbons and degradation process. Reproduced with permission.^[^ ^114^ ^]^ Copyright 2015, American Chemical Society.

Gelatin is a natural protein obtained by acid or alkaline hydrolysis of collagen (based on anion and cation groups in the gel network).^[^ ^51^ ^]^ It is widely used due to its advantages, including nontoxicity, high water absorbency, biocompatibility, and biodegradability.^[^ ^52^, ^53^, ^54^ ^]^ The simplest method to prepare gelatin gel is to mix gelatin powder with hot water. When the temperature drops to the sol‐gel transition temperature, the gelation process begins during cooling. This gel tends to fracture under stretching and rapidly dries under ambient conditions, like most hydrogels, due to water evaporation, which hardens and shrinks the material. A multifunctional biogel based on cellulose, gelatin, and citric acid for pneumatic actuators and multifunctional sensors was prepared by Baumgartner et al.,^[^ ^55^ ^]^ as shown in **Figure** **2**b. The gelatin‐based biogel could monitor temperature, humidity, and stress. Moreover, the biogel possessed degradable properties, allowing it to be enzymatically degraded by wastewater bacteria within five days.

Chitosan, a natural cationic polymer derived from the deacetylation of chitin,^[^ ^56^ ^]^ is distinguished by excellent biocompatibility, biodegradability, and non‐toxicity, rendering it an ideal material for biomedical applications.^[^ ^57^, ^58^ ^]^ It is insoluble in water and alkaline solutions but soluble in acidic conditions. Specifically, chitosan can be degraded by lysozyme and chitosanase. These biodegradation properties of chitosan make it promising for wound healing, drug delivery systems, and as a scaffold material in tissue engineering.^[^ ^59^, ^60^ ^]^ Nan et al. fabricated a bioinspired multifunctional soft actuator by chitosan and magnetic nanoparticles (MNPs), designed in shapes such as flowers, leaves, larvae, and fingers, as shown in Figure 2c.^[^ ^61^ ^]^ The flexible actuator was capable of responding to various stimuli, such as humidity, temperature, and magnetic fields. The characteristics enabled movements such as locomotion and grasping. The actuator demonstrated potential as a wireless miniature robot in biomimetic and biomedical applications. Moreover, the actuator exhibits degradable properties over 28 days. The shape of the multifunctional chitosan‐MNP soft actuator progressively diminished over time, ultimately leaving only the MNPs visible.

Silk fibroin is a natural biopolymer material derived from silk, primarily composed of amino acids such as glycine (Gly), alanine (Ala), serine (Ser), and small amounts of other amino acids, such as tyrosine, leucine, and glutamic acid.^[^ ^62^ ^]^ Silk fibroin exhibits excellent biocompatibility, enabling it to integrate well with biological tissues without inducing strong immune responses.^[^ ^63^ ^]^ The biodegradation rate of silk fibroin can be controlled through chemical or physical modifications, ranging from a few minutes to several days.^[^ ^64^ ^]^ The properties of silk fibroin can be improved through modifications, such as incorporating nanoparticles or compositing with other materials, to enhance its properties.^[^ ^65^, ^66^ ^]^ Based on these numerous advantages, silk fibroin has found extensive applications in the biomedical field,^[^ ^67^ ^]^ textile industry,^[^ ^68^ ^]^ and food industry.^[^ ^69^ ^]^ Recently, silk fibroin has also been widely employed in the preparation of flexible sensors and bioelectronic devices, such as serving as a substrate for implantable devices^[^ ^70^, ^71^, ^72^ ^]^ and a stretchable sensor as artificial electronic skin.^[^ ^73^ ^]^

In the field of biodegradable synthetic plastics, polylactic acid (PLA) is an aliphatic polyester derived from renewable resources such as starch and sugar.^[^ ^74^ ^]^ It is compatible with the biological tissue.^[^ ^75^ ^]^ PLA has been widely applied in various biomedical fields,^[^ ^76^ ^]^ for example, a graphene oxide (GO)‐incorporated polylactic acid (PLA) (GO‐PLA) films was fabricated for guided bone regeneration.^[^ ^77^ ^]^ Its Young's modulus is 2–3.5 GPa and its strain‐to‐failure is 4%–7%. However, the low glass transition temperature (58 °C) limits its use in broader applications. Chemical modification of L and D lactic acid isomers of PLA leads to the formation of lactide, which polymerizes into various poly‐L‐lactic acid (PLLA) and pure poly‐D‐lactic acid (PDLA) forms.^[^ ^75^ ^]^ These hydrogels are also biocompatible and biodegradable, and have higher Young's moduli (4 GPa) and slower degradation compared with PLA. The crystallinity and orientation of PLLA can be altered through thermal treatment and mechanical stretching, thereby imparting piezoelectric properties and making it suitable for use in sensors. PLLA has been used to create a flexible pressure sensor^[^ ^78^ ^]^ and a piezoelectric sensor for the evaluation of motor function recovery after nerve injury.^[^ ^79^ ^]^ The disadvantages of PLA, PLLA, and PDLA are slow degradation rate, hydrophobicity, and low impact resistance during use.^[^ ^75^ ^]^

Polyvinyl alcohol (PVA) is a widely used material produced from polyvinyl acetate through hydrolysis or alcoholysis reactions. Its molecular chain contains a large number of hydroxyl groups, which confer unique hydrophilicity and chemical reactivity. It is a water‐soluble polymer capable of forming flexible layers. PVA exhibits excellent physical properties, including high tensile strength, flexibility, film‐forming ability, and adhesion. Due to its biocompatibility, PVA is also utilized in the biomedical field, for example, drug delivery systems^[^ ^80^, ^81^ ^]^ and artificial cartilage.^[^ ^82^, ^83^ ^]^ In the field of biodegradable electronic devices, PVA can be used as a biodegradable substrate^[^ ^31^ ^]^ and encapsulation layer,^[^ ^84^ ^]^ such as in temporary implantable devices.^[^ ^85^ ^]^ Due to its high insulating properties, PVA is also applicable as a dielectric layer for capacitors or transistors.^[^ ^86^ ^]^

Poly‐caprolactone (PCL) is a biodegradable semi‐crystalline polymer. The mechanical properties of PCL primarily depend on its molecular weight, crystallinity, and porosity. Compared to other biodegradable materials, its elastic modulus is relatively high, ranging from 0.21 to 0.44 GPa,^[^ ^87^ ^]^ with a relative small 5%–10% strain until yielding. PCL is typically degraded by enzymes or fungi, requiring 1 to 2 years.^[^ ^87^ ^]^ However, under physiological conditions, due to the lack of suitable enzymes, PCL undergoes negligible degradation, with a degradation time range of 2 to 4 years.^[^ ^88^ ^]^ To improve the degradation rate and mechanical properties of PCL, it is commonly blended with other lactide‐based materials such as PLA, PLLA, PLGA, and polyethers.^[^ ^89^, ^90^ ^]^ Similarly, Poly(l‐lactide‐co‐ε‐caprolactone) (PLCL) is a biodegradable ester‐based polymer. It has good flexibility, controlled degradation rate, and exhibits a balance between hydrophobicity and biocompatibility. Han et al.^[^ ^91^ ^]^ fabricated a super‐elastic elastomer, poly(l‐lactide‐co‐ε‐caprolactone) (PLCL), composed of poly(L‐lactide‐co‐ε‐caprolactone). The elastomer could be used in the fabrication of soft robotic grippers and suture‐free cardiac jackets with bio‐inspired designs, achieving applications in soft robots and biomedical implants. The elastomer exhibited degradable properties, with the degradation process shown in Figure 2d. When the PLCL films (140k) half‐immersed in phosphate buffer solution (PBS, pH 7) at 37 °C, the membrane gradually degraded through hydrolysis without remaining any toxic byproducts, and completely disappeared at 30 weeks.

The polymers reported above possess dielectric properties and are primarily used as substrates, encapsulants, or adhesives in sensors. However, in many sensors, conductive materials are required as electrodes. Conductive polymers offer a series of exploitable advantages in biodegradable sensors, including biocompatibility, degradability, and ease of preparation, with the ability to be processed at low temperatures. PPy is an important conductive polymer formed by the chemical or electrochemical polymerization of pyrrole monomers.^[^ ^92^ ^]^ It exhibits excellent conductivity, flexibility, and mechanical properties, making it suitable for flexible electronic devices. The conductivity of PPy can be adjusted by doping with different ions (e.g., Cl^−^, SO_4_^2−^),^[^ ^93^, ^94^ ^]^ enabling its application in various electronic devices. Additionally, PPy demonstrates good biocompatibility, offering potential applications in biomedical electronic devices,^[^ ^95^ ^]^ soft sensors,^[^ ^96^, ^97^, ^98^ ^]^ supercapacitor^[^ ^99^ ^]^ and e‐skins.^[^ ^100^, ^101^ ^]^ PPy can degrade slowly in natural environments, and can also be combined with other biodegradable polymers, but its biodegradability has not yet been widely recognized. Poly(3,4‐ethylenedioxythiophene) (PEDOT) also has excellent charge transport capabilities and chemical stability, making it a commonly used conductive polymer.^[^ ^102^ ^]^ While PEDOT itself is not biodegradable, its biodegradability can be improved by combining it with biodegradable polymers, such as montmorillonite,^[^ ^103^ ^]^ carboxymethyl chitosan^[^ ^104^ ^]^ and silk proteins.^[^ ^105^ ^]^ It is synthesized from aniline monomers through either chemical or electrochemical polymerization. The resulting structure consists of repeating aniline units and a conjugated system, which imparts excellent conductivity to the material.^[^ ^106^, ^107^ ^]^ The conductivity of PANI can be modulated through doping. In its undoped state, PANI exhibits poor conductivity; however, acid doping (e.g., sulfuric acid, hydrochloric acid) significantly enhances its conductivity.^[^ ^108^ ^]^ Additionally, PANI demonstrates remarkable environmental stability, resisting acids, bases, moisture, and oxidation. Its applications span across sensors (such as gas sensors^[^ ^109^ ^]^ and pH sensors^[^ ^110^ ^]^), supercapacitors,^[^ ^111^, ^112^ ^]^ infrared electrochromic devices. Similar to PPy and PEDOT, while it exhibits good biocompatibility, its biodegradability is notably poor.

#### 2.2.3 Inorganic Semiconductor and Dielectric

Si, SiO_2_, and Si_3_N_4_ are widely used materials in electronic devices, compatible with traditional semiconductor processes, and are typically employed in high‐performance electronic devices. When in nanoform, they can fully dissolve in biological fluids.^[^ ^35^, ^36^ ^]^ Si, being a semiconductor, is commonly used as a functional material in electronic devices. In contrast, SiO_2_ and Si_3_N_4_ often serve as the encapsulation layer because of their slow degradation.^[^ ^113^ ^]^ Their degradation rates depend on the surrounding solution, temperature, pH, and deposition conditions. The hydrolysis of them is extensively researched, and their dissolution in biofluids is presented as follows:

(3) $$\mathrm{Si}+4H_{2}O\to \mathrm{Si}{\left(\mathrm{OH}\right)}_{4}+2H_{2}↑$$

(4) $$\mathrm{Si}O_{2}+2H_{2}O\to \mathrm{Si}{\left(\mathrm{OH}\right)}_{3}$$

(5) $$Si_{3}N_{4}+6H_{2}O\to 3\mathrm{Si}O_{2}+4NH_{3}↑$$

(6) $$Si_{3}N_{4}+12H_{2}O\to 3\mathrm{Si}{\left(\mathrm{OH}\right)}_{4}+4NH_{3}$$

Hwang et al.^[^ ^114^ ^]^ fabricated a biodegradable pH sensor based on doped nanoribbons of silicon (Si NRs) functionalized with 3‐aminopropyltriethoxysilane. The electrodes and interconnects were served by Mg, the interlayer dielectrics and encapsulants were served by SiO_2_, and the substrate was served by poly(1,8‐octanediol‐co‐citrate) (POC). The device enabled real‐time monitoring of aqueous solution pH through dynamic electrical conductivity variations. It demonstrated the promising applicability in biomedical sensing technologies. Particularly, when the pH sensor was immersed in aqueous solution, the Mg electrodes completely disappeared after 12 h via reactive dissolution (i.e., hydrolysis). Moreover, Si, SiO_2_, and POC degraded in several weeks.

## 3 Sensors of Soft Robotics

The principle of a sensor is to detect physical quantities in the environment through sensitive components (such as pressure, temperature, humidity, light intensity, etc.) and convert them into measurable electrical signals (such as voltage, current, resistance, capacitance, inductance, or impedance).

### 3.1 Sensing Principles

**Figure 3:**
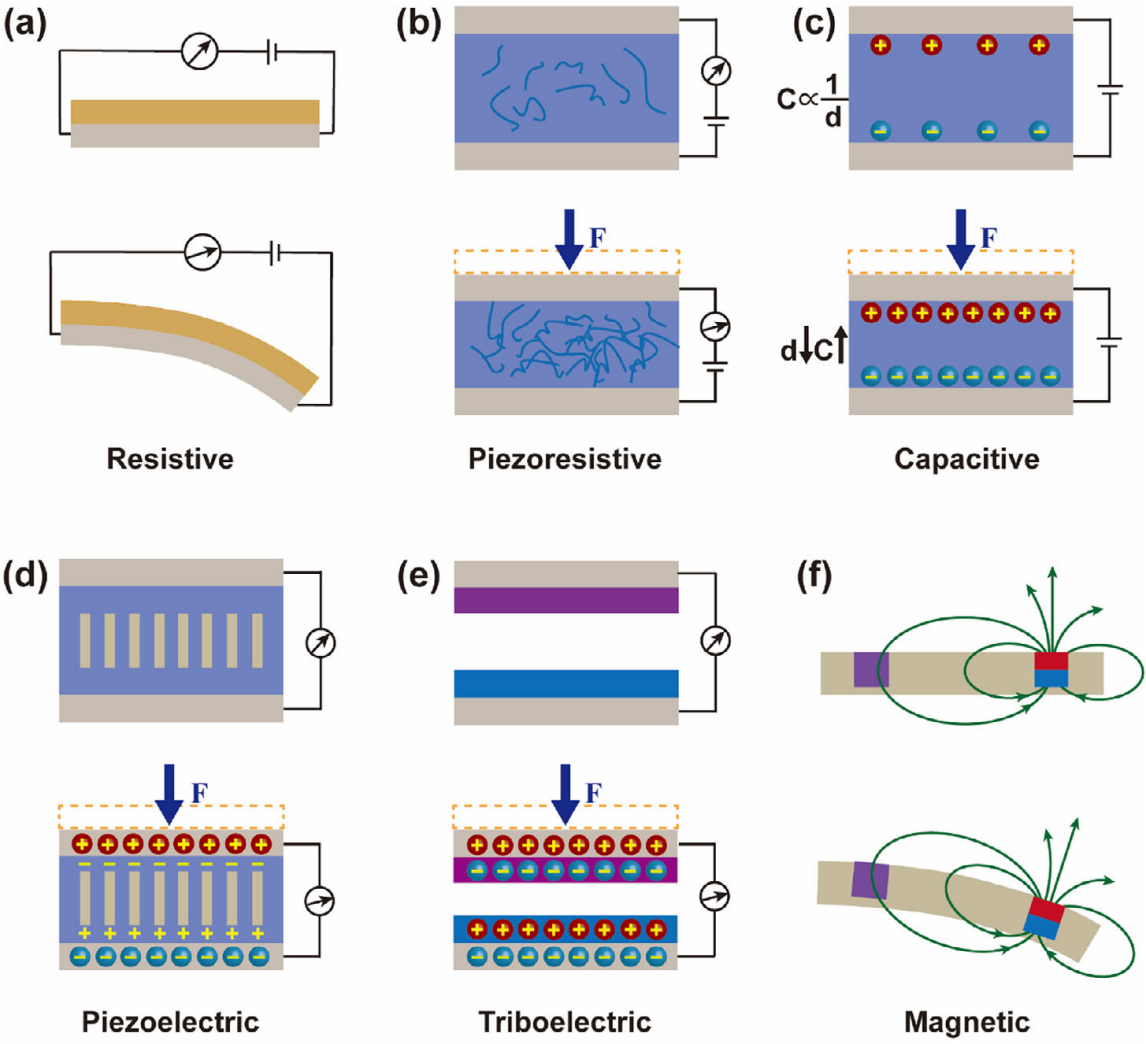
The main working mechanism of sensors. a) Resistive sensing. b) Piezoresistive sensing. c) Capacitive sensing. d) Piezoelectric sensing. e) Triboelectric sensing. f) Magnetic sensing.

Resistive Sensing: Resistive sensing is based on the characteristic that the resistance value changes with the external effect. The resistance of elastic conductive materials in soft robots varies with their deformation states, as shown in **Figure** **3**a. Resistance depends on the parameters given in Equation (6).

(7) $$R=\rho \frac{l}{A}$$

Here, 𝜌 is sheet resistivity, L is the length of the material, and A is the area. Any external effect, such as strain, stress, pressure, causes a variation of one or more of these parameters, resistance will be subjected to change. This sensor allows soft robots to gain information about their surroundings through contact‐based interactions.

Piezoresistive sensing: Piezoresistive sensors operate based on the piezoresistive effect. They convert pressure signals into electrical signals by utilizing the change in resistance of piezoresistive materials under pressure. The working mechanism of piezoresistive sensors is shown in Figure 3b. This change in resistance is determined by the physical properties of the piezoresistive materials. When pressure is applied to the piezoresistive materials, the crystal lattice structure of the material undergoes slight changes, leading to variations in its conductivity and consequently in its resistance value. Under ideal conditions, after being processed by signal conditioning circuits, these signals should ideally output an electrical signal proportional to the pressure. However, in experimental applications, the change in resistance of piezoresistive materials is typically small, and its sensitivity and stability may be influenced by factors such as temperature, humidity, and others. Additionally, the processing and manufacturing of materials and sensors are crucial, as excellent materials and processes can result in better and more stable sensor performance.

Capacitive Sensing: A capacitive sensor is a device that detects changes in its surroundings by sensing variations in capacitance. Its basic structure consists of two conductors and a dielectric material arranged in a sandwich configuration. When an external force, such as pressure or strain force, acts upon the sensor, causes changes in the electrodes of the capacitive sensor, the capacitance value undergoes corresponding changes. The working mechanism is shown in Figure 3c. As indicated in Equation (7).

(8) $$C=\varepsilon_{r}\varepsilon_{0}\frac{A}{d}$$

In Equation (7), ε_*r*_ is the dielectric constant of the medium between surfaces, ε_0_ is the dielectric constant of air, A is the area of the facing plates, and d is the distance between these plates. C is a measure of the ability of a system to store an electric charge, and it is directly proportional to A, and the *ε*_r_ *ε*_0_, while inversely proportional to the d between the electrodes. So, when the electrode surfaces are bendable, stretchable, or movable in soft sensor applications, the capacitance will change with the change in the area, distance, or dielectric separator. Their sensitivity and measurement range can be optimized by adjusting structural parameters or selecting different dielectric materials. Unlike piezoelectric sensors, capacitive sensors operate based on capacitance changes, making them suitable for measuring static or slowly varying signals.

Piezoelectric Sensing: Piezoelectric sensors operate based on the piezoelectric effect. The piezoelectric effect refers to the phenomenon where certain materials generate charges on their surfaces when subjected to mechanical stress or deform under the influence of an electric field, as shown in Figure 3d. Specifically, when piezoelectric materials are subjected to external forces, the internal crystalline lattice structure deforms, causing a relative displacement of positive and negative charge centers. This creates a voltage difference on the material's surface, resulting in a voltage output. This process effectively converts applied strain or pressure into electrical signals. Piezoelectric sensing is characterized by high sensitivity, fast response, and a wide bandwidth, making it widely used in applications such as pressure sensing and acceleration sensing. However, piezoelectric sensors are susceptible to temperature and humidity, and in some cases, the sensor's output signal may not maintain a strict linear relationship with the input signal.

Triboelectric Sensing: Triboelectric sensing is based on the triboelectric effect.^[^ ^115^, ^116^, ^117^ ^]^ When two dissimilar materials come into contact and rub against each other, electrons transfer from one material to the other due to differences in their electron affinities, generating static charges. The principle is shown in Figure 3e. Upon separation, an electrical potential difference forms between the materials due to the electrostatic field. When connected to an external circuit, this potential difference drives electron flow, producing a measurable current signal.^[^ ^118^ ^]^ Different material pairs significantly influence charge transfer efficiency and sensor sensitivity. For instance, PTFE and metal combinations may generate stronger signals than polymer‐polymer pairs. Additionally, surface microstructure design, such as nanoscale textures, can enhance charge transfer by increasing contact area, thereby improving sensor performance.

Magnetic Sensing: Magnetic sensors are devices used to detect changes in magnetic fields, operating on the principle of the physical response of materials to magnetic fields. Common types include Hall effect sensors, magnetoresistive sensors, and magnetic tunnel junction sensors. Hall effect sensors generate a voltage due to the deflection of charge carriers in a magnetic field, while magnetoresistive sensors detect magnetic fields by utilizing changes in resistance caused by the magnetic field. The working mechanism of magnetic sensors is shown in Figure 3f. Magnetic sensors typically employ semiconductor materials such as gallium arsenide and indium antimonide, which exhibit significant Hall effects in magnetic fields, enhancing the sensitivity of sensors. Magnetoresistive sensors may utilize magnetic materials, such as ferromagnetic materials, to improve magnetic field sensitivity. Unlike resistive sensors, magnetic sensors are cost‐effective and do not suffer from hysteresis, but they are susceptible to external effects from metals or magnets.

### 3.2 Biodegradable Sensors

In sensor systems, degradation can be triggered by both active and passive mechanisms. Among them, active degradation triggers often arise from external environmental conditions such as temperature and humidity fluctuations, or light exposure. For example, high temperatures can accelerate material aging or cause phase transitions, leading to performance degradation. Similarly, moisture ingress may facilitate chemical reactions like hydrolysis, affecting the stability of sensitive materials.

In contrast, the occurrence of passive degradation mechanisms does not require external triggers. These degradation processes typically arise from the inherent physical or chemical properties of the material, its structural stability, and the interactions between different materials. Passive degradation mechanisms are often controlled by isolating the reaction conditions or reactants, as they are closely linked to the design and selection of the materials themselves. Therefore, understanding these mechanisms is crucial in the design and manufacture of sensor systems, as it plays a key role in improving the long‐term stability of both materials and devices. For example, protective coatings or encapsulation techniques can slow down the degradation of organic optoelectronic devices by shielding them from moisture, oxygen, or UV radiation. These methods enhance the durability of materials and the overall lifespan of sensor systems. From another perspective, active degradation mechanisms enable flexible robots to undergo controllable degradation at specific times, thereby improving recyclability and promoting environmental sustainability in certain applications.

By considering both active and passive degradation triggers, we can better understand the longevity and reliability of sensor systems, which is crucial for designing devices that maintain performance over extended periods of use. In this section, we classify the sensors based on their functions and application scenarios, and discuss the output performance and degradation mechanisms of stress sensors, environmental sensors, olfactory sensors, and bio‐implantable sensors.

#### 3.2.1 Tactile Sensor and Strain/Pressure Sensor

Tactile perception is one of the most important feedback mechanisms for robots interacting with their environment. Tactile sensors detect contact forces, pressure distributions, or deformations, enabling soft robots to perceive the position, shape, and rigidity of external objects. This capability facilitates precise grasping, obstacle avoidance, and collaborative manipulation. Since most traditional sensors are designed for rigid structures, integrating them into the flexible and agile bodies of soft robots presents significant challenges. Soft robots theoretically possess infinite degrees of freedom (DOFs), meaning sensors used to measure their continuous posture changes must have a sufficient range of motion, accuracy, and resolution. Developing high‐performance, biodegradable soft sensors for soft robotics represents a long‐standing challenge. The mechanisms of tactile sensors are transducing applied pressure into electrical signals.^[^ ^119^ ^]^ Most widely used mechanisms employed in tactile sensors include resistive, piezoresistive, capacitive, piezoelectric, and triboelectric types. However, no matter what kind of mechanisms are used by the tactile sensors, each tactile sensor has a sandwich structure, two electrodes with a dielectric intermediate layer, and (if required) a substrate or encapsulation.

A fully biodegradable, ultra‐sensitive crack‐based flexible resistive strain sensor was fabricated using a multilayer approach with Mo film, MoO_3_ adhesion layer, PCL substrate, and wax‐based polymeric composite (Cw/Bw/PBAT) encapsulation layer.^[^ ^120^ ^]^ The results of in vivo Real‐Time Hemodynamic Monitoring by this sensor confirmed the efficacy of ultrasensitive, temporal, and continuous monitoring with soft and conformal contact on tissues. The dissolution test of the encapsulated Mo/MoO_3_/PCL crack‐based strain sensor immersed in 1 M PBS at pH 7.4, 37 °C, showed that the device began to dissolve Mo trace due to water permeation through the encapsulation membrane (2 weeks) and continuously degraded of remained polymeric layers into small debris (4 and 6 weeks), implying no need for removal surgery due to its biodegradation properties after implantation.

A biodegradable Ecoflex encapsulated bacterial cellulose/polypyrrole strain resistive sensor was fabricated by Gao and co‐workers.^[^ ^121^ ^]^ This strain sensor, prepared by in situ fermentation with PPy and encapsulation with Ecoflex, exhibiting high sensitivity (gauge factor of 3.21–4.86), large strain ranges (up to 90% strain), ultralow strain detection limit (0.05%) and remarkable long‐term stability without any distinct decline in sensitivity after a constant applied stretching of 90% for 1000 cycles. The biodegradability of this sensor was tested in the natural soil environment, where the soil moisture was around 11–14 (% vol) and the soil temperature was around ≈20 °C. The complete degradation of the sensor took about 90 days, with one piece of residue left. The low degradation rates under natural conditions might be introduction of Ecoflex. Replacing Ecoflex with bio‐derived encapsulation materials that have shorter and adjustable degradation will further enhance the environmental friendliness of these devices.

Silk fibroin (SF) and reduced graphene oxide are both biodegradable materials, that can be used in various devices. By incorporating rGO into silk fibroin, a rGO/SF composite hydrogel with improved conductivity and mechanical properties could be used to fabricate resistive pressure sensors and stretch sensors, and both show excellent sensing performance.^[^ ^122^ ^]^ The rGO/SF hydrogel had good biodegradability, which could be fully degraded not only in 0.1 m NaOH within 28 h, but also in DI water within 60 days.

**Figure 4:**
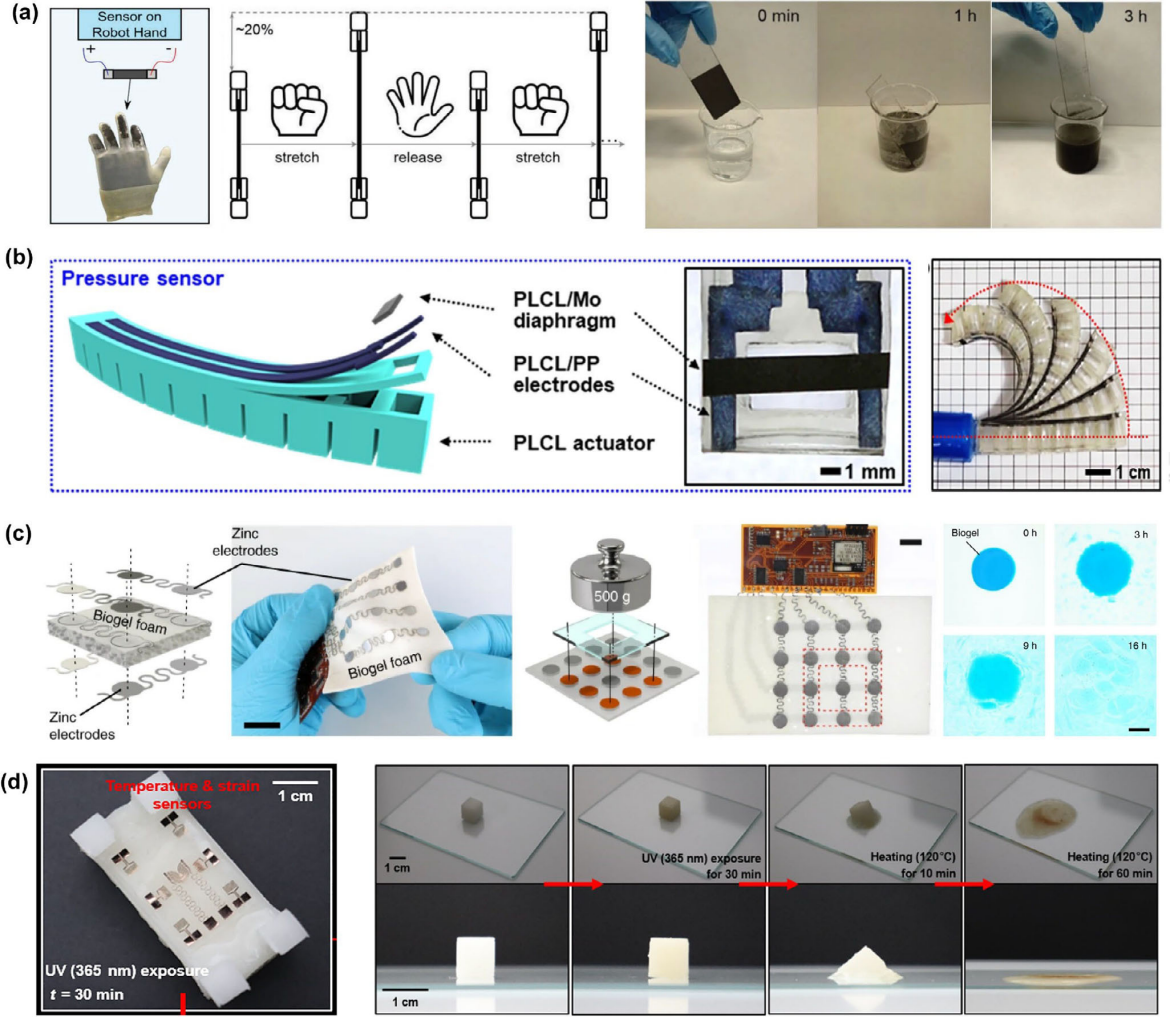
Pressure and strain sensor based on various degradable materials. a) Schematic diagram of the biodegradable vitrimer conductive coating‐based strain sensor for a robot hand and the biodegradable process. Reproduced with permission.^[^ ^126^ ^]^ Copyright 2023, Wiley. b) Structure of PLCL‐based pressure sensor and image of soft robot in bending state. Reproduced with permission.^[^ ^91^ ^]^ Copyright 2023, Springer Nature. c) Schematic of the capacitive pressure sensor array using biogel foam as the dielectric layer, and the degradation profile of the gel. Reproduced with permission.^[^ ^131^ ^]^ Copyright 2023, Springer Nature. d) Schematic diagram of stretch sensor and DPI‐HFP/Ecoflex 00–30 degradation process based on the DPI‐HFP/Ecoflex 00–30 soft gait robot. Reproduced with permission.^[^ ^133^ ^]^ Copyright 2023, American Association for the Advancement of Science.

The piezoresistive sensor is a type of resistive sensor, specifically focused on sensing the resistance change induced by pressure.^[^ ^123^ ^]^ Moreover, degradable materials can endow sensors with more diverse functions. For example, Wang et al. fabricated a bioinspired supramolecular hydrogelation‐based tactile sensor with intrinsic biocompatibility and biodegradability.^[^ ^124^ ^]^ A tactile sensor with a 3 × 3 piezoresistive sensing array was fabricated with the 9‐fluorenylmethoxycarbonyl‐modifid diphenylalanine (Fmoc‐FF)‐based self‐assembled nanoribbons and nanofibers (doped with PEDOT:PSS) hydrogel, which behaved as the encapsulation layer and sensing units, respectively. The ∆*R/R_0_* evolution curve of this tactile sensor exhibited two‐segmented slopes of *S1 *= 5.7% kPa^−1^ and *S2 *= 0.5% kPa^−1^ when the pressure was lower and higher than 8.0 kPa, respectively, with a detection limit ranging from 0.4 to 15.0 kPa. Fmoc‐FF hydrogel had good biocompatibility and rapid biodegradability. Pan et al. reported a biodegradable multilayer piezoresistive pressure sensor composed entirely of biodegradable materials: natural leaf veins, poly(lactic‐co‐glycolic acid) (PLGA), and polyvinyl alcohol (PVA) nanofiber films.^[^ ^125^ ^]^ The PLGA and PVA nanofiber films were fabricated using electrospinning technology and integrated with natural leaf veins, forming a 3D porous hierarchical structure of the sensor. This unique architecture endows the sensor with excellent biodegradability, high breathability, and superior pressure sensitivity. The biodegradability of the sensor was tested in a PBS solution (pH 7.4), the whole device loss about half of its weight after 45 days. Spallanzani et al. developed a piezoresistive strain sensor based on a biodegradable vitrimer conductive coating.^[^ ^126^ ^]^ By applying the coating to the surface of a robotic SoftHand3, the bending state of the hand could be inferred through changes in electrical resistance. The sensor demonstrated excellent stretchability, with a strain capacity of up to ≈1000%. The relative resistance (*R*/*R*₀) exhibited a nonlinear relationship with applied strain, increasing monotonically as the strain increased. As shown in **Figure** **4**a, the glass vitrimer conductive coating could be completely dissolved after immersion in 90% ethanol for 3 h, indicating good biodegradability. Han et al. developed a piezoresistive pressure sensor using the biodegradable material poly(L‐lactide‐co‐ε‐caprolactone) (PLCL).^[^ ^91^ ^]^ The sensor measured pressure by monitoring resistance changes under different loading conditions. It exhibited a high sensitivity and was capable of detecting pressure distributions as low as a 10 mg cotton ball, demonstrating excellent resolution. The device functioned reliably under various external conditions and maintained performance even under repeated bending. As shown in Figure 4b, the pressure sensor was integrated at the tip of a transient soft robotic gripper for real‐time pressure monitoring during object manipulation.

Using self‐healing materials to fabricate soft robotics is significant, as it can extend robotic lifespan and reduce material waste. For certain soft robotics, such as those used in drug delivery, this approach alone is insufficient. These soft robotics additionally require biodegradability to eliminate the need for surgical removal procedures after the complete mission. However, few soft robotics systems with all components that have the properties of both biodegradable and self‐healing have been reported. For the first time, Hwang et al. introduced a stretchable, biodegradable, self‐healing conductor and a biodegradable and self‐healing elastomer to build a biodegradable multifunctional electronic device.^[^ ^127^ ^]^ The conductor composed of PEDOT:PSS, doped with biocompatible additives for enhanced mechanical and electrical properties. Poly(lactide‐*co*‐ε‐caprolactone)‐diol (PLCL‐diol) served as a soft segment of the elastomer. The conductor and the elastomer were used to fabricate a multifunctional electronic tree with humidity, temperature, and capacitive pressure sensors. The biodegradable and self‐healing properties demonstrated their great application potential in sustainable soft robots.^[^ ^128^, ^129^ ^]^

By utilizing cellulose and pectin as main components, an edible and biodegradable capacitive pressure sensor was fabricated.^[^ ^130^ ^]^ The sensor was fabricated in a sandwich structure where a porous pectin xerogel was positioned between two cellulose substrates, with the carbon paste‐coated side oriented toward the xerogel. The sensor exhibited a range of desirable properties, including moderate sensitivity (0.0294 kPa^−1^), rapid response time (118 ms), and excellent durability over 10000 loading/unloading cycles. The sensor materials possessed the ability to naturally degrade over time, thereby minimizing environmental impact. Baumgartner et al. developed a capacitive pressure sensor using a biodegradable bio‐gel as the dielectric layer, which enabled sensitive detection of pressure variations.^[^ ^131^ ^]^ The sensor was capable of detecting pressures ranging from 0 to 102 kPa. Notably, the dielectric layer could be fully degraded in DI water within 16 h. As shown in Figure 4c, the sensor array was capable of recognizing objects with complex shapes. Under ambient conditions, the sensor maintained functional stability for over one year. Furthermore, the sensor was integrated at the tip of a soft pneumatic robot resembling an elephant's trunk to monitor the pressure exerted during object manipulation. Oh et al. developed a fully biodegradable transient soft robot based on a DPI‐HFP/silicone elastomer composite material. The robot integrated a capacitive strain sensor whose capacitance varies with the bending angle of the robot. Under cyclic pneumatic actuation between −10 and 10 kPa, the capacitance consistently changed by 1 pF, demonstrating stable sensor performance. As shown in Figure 4d, the robot could be completely dissolved after exposure to 365 nm UV light for 30 min followed by thermal treatment at 120 °C for 1 h, indicating excellent controllable degradability.

A highly biodegradable tactile sensor was fabricated by using β‐glycine‐gelatine ferroelectric composite.^[^ ^132^ ^]^ The sensor was made of two pieces of one‐sided patterned glycine‐gelatine composite films, with patterned surfaces oriented toward each other. The fabricated tactile sensor exhibits excellent piezoelectric sensitivity, reaching 41.3 ± 1.3 mV kPa^−1^, with a fast response time of 1.0 ± 0.1 ms. The biodegradability of the sensor was tested in normal tap water. The complete degradation of the sensor took about a few days, leaving only the traces of the Au electrode. Replaced the Au electrode by a biodegradable metal, such as Mg or Zn, the sensor will be fully biodegradable.

**Table** **2** compares tactile sensors and strain/pressure sensors with different sensing strategies, materials, performance, and degradation time. When sensors employ different sensing strategies, they exhibit distinct performance even if constructed from the same material, as shown in Table 2. Since sensors are composed of several distinct components, and each component may utilize one or more different materials, the degradation rates of these materials may be different. Consequently, the time of performance and degradation of the sensors is determined by the fastest‐degrading and slowest‐degrading materials, respectively. The fastest‐degrading material dictates the operational lifespan of the sensor, while the slowest‐degrading material determines the full degradation time of the sensor.

**Table 2:** Sensing strategies, materials, performance, and degradation of tactile sensor and strain/pressure sensor.

Sensing Strategies	Materials	Sensitivity	Detection limit	Mechanical flexibility	Response time	Degradation time	Refs.
Resistive	Mo, MoO_3_, PCL	gauge factor of 1355 at 1.5% strain	0.5%	strains of 10%	1–10 s	6 weeks in PBS (1 M, pH 7.4, 37 °C)	[120]
Resistive	bacterial cellulose, PPy, Ecoflex	gauge factor of 3.21–4.86	0.05%	strain ranges: up to 90%	71 ms	90 days in soil with one piece of residue	[121]
Resistive	Silk fibroin, rGO	0.4 kPa^−1^	100 Pa–500 kPa		160 ms	within 60 day in DI water	[122]
Piezoresistive	Fmoc‐FF, PEGDA, PEDOT:PSS	0.5% kPa^−1^	0.4–15.0 kPa		0.28 s	7‐day of implantation in mice	[124]
Piezoresistive	PLGA, PVA	6.33 kPa^−1^	0.03–11.60 kPa		161 ms	loss half of its weight after 45 days in a PBS solution (pH 7.4)	[125]
Piezoresistive	vitrimer, graphene nanoplatelets, carbon nanofibers	relative resistivities of 258.00	1%–200%	200% of stretch		3 h in 90% ethanol	[126]
Piezoresistive	PLCL, PEDOT:PSS, Mo	gauge factors of 5500–7500	≈0.01%	≈1600% of stretch		30 weeks in a PBS solution (pH 7)	[91]
Capacitive	PLCL‐diol, PEDOT:PSS			≈500% of stretch		lose 70%–90% of its weight after 10 weeks in a PBS solution (RT, pH 7)	[127]
Capacitive	cellulose, carbon paste, pectin	0.0294 kPa^−1^	10 Pa–100 kPa		118 ms	≈112 day in Fungi	[130]
Capacitive	gelatin		0–102 kPa	strain of 300%		lose ≈90% of its weight after 14 days in wastewater	[131]
Capacitive	DPI‐HFP, silicone composites (Sylgard‐184 and Ecoflex 00–30)	1 pF	−10–10 kPa			≈1.5 h, controlled by 365 nm UV light the followed by thermal treatment	[133]
Piezoelectric	β‐glycine‐gelatine	41.3 ± 1.3 mV kPa^−1^	2.5–55 kPa		1.0 ms	7 days in tap water	[132]

#### 3.2.2 Environmental Sensors

**Figure 5:**
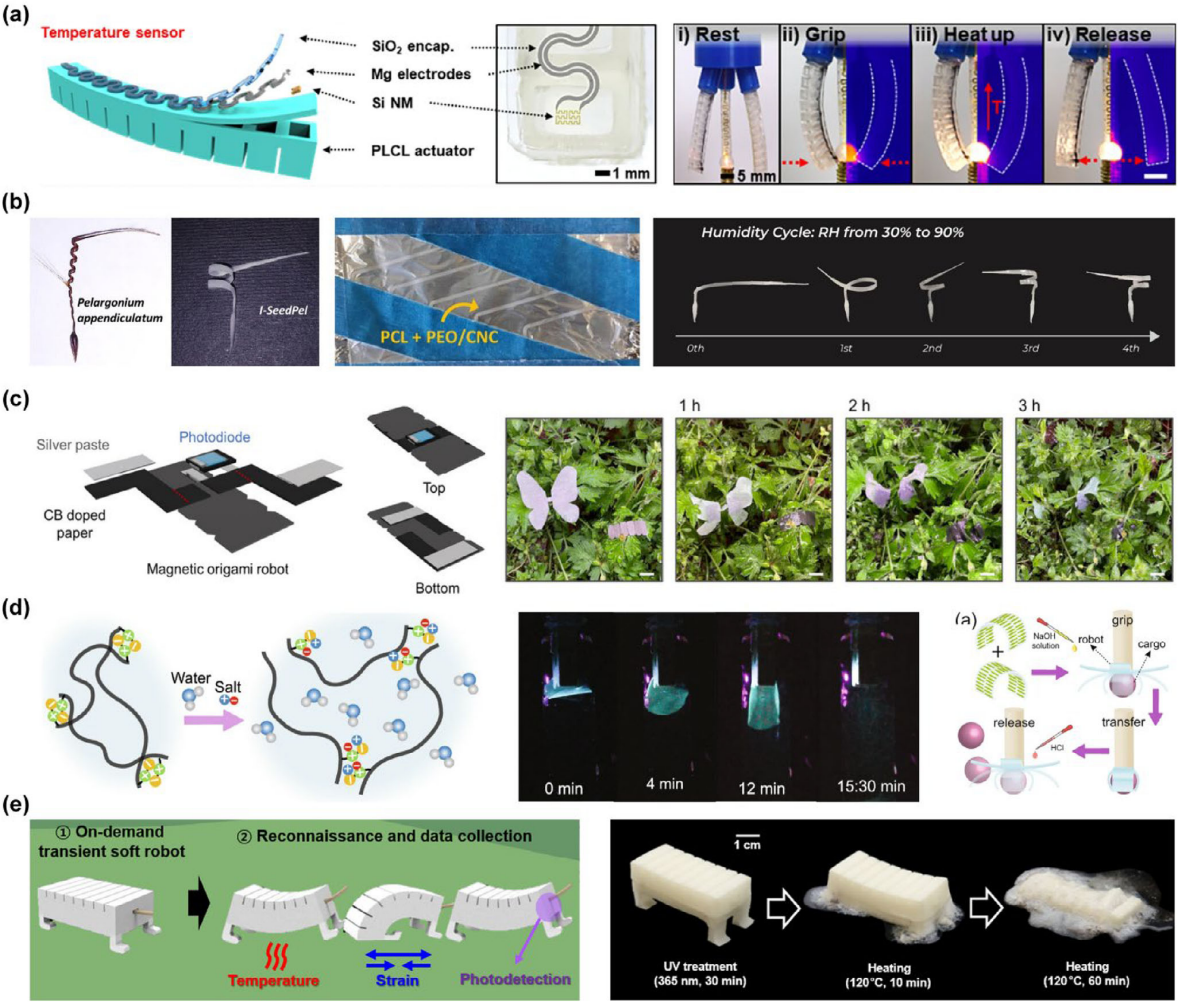
Biodegradable soft robots with integrated environmental sensors (humidity, temperature, light, and pH). a) Schematic illustration of a PLCL‐based transient robotic gripper that perceives environmental factors, such as temperature, through integrated electronic components, and the overall procedures of temperature‐aware actuation illustrate the sequence of i) rest, ii) grip, iii) heat up, and iv) release. Reproduced with permission.^[^ ^91^ ^]^ Copyright 2023, Springer Nature. b) Biomimetic I‐SeedPel fabricated by biodegradable polymers demonstrating deformation under consecutive humidity cycles. Reproduced with permission.^[^ ^136^ ^]^ Copyright 2023, Elsevier. c) Schematic illustration of light‐sensing paper robots, accompanied by photographs depicting the degradation of magnetic paper robots in nature through contact with water. Reproduced with permission.^[^ ^138^ ^]^ Copyright 2024, John Wiley & Sons. d) The on‐demand degradation of physically crosslinked hydrogel with a 3:1 DMAPS:MAA weight ratio (GelWC) in a 10 wt.% NaCl solution and the schematic illustration of the pH‐responsive micro‐gripper. Reproduced with permission.^[^ ^139^ ^]^ Copyright 2023, Springer Nature. e) Illustration of a lifetime configurable gaiting robot capable of conducting reconnaissance and data collection through integrated sensors for temperature, strain, and photodetection (left) and time‐lapse image (right) of the gaiting robot undergoing decomposition at 120 °C for 30 min after exposure to UV light (365 nm). Reproduced with permission.^[^ ^133^ ^]^ Copyright 2023, American Association for the Advancement of Science.

In addition to pressure and strain, some soft robots, such as those used for drug delivery, also require data on temperature, humidity, and other factors to fully comprehend their environment.^[^ ^134^ ^]^ Metal‐based temperature sensors are among the earliest developed temperature sensors, utilizing the temperature‐dependent resistance of metallic materials for measurement. Their core component is the temperature‐sensitive element, typically made from pure metals or specific alloys, such as platinum (Pt), gold (Au), nickel (Ni), and manganese (Mn). A deformable temperature sensor using serpentine Mg traces encapsulated in Ecoflex was reported.^[^ ^38^ ^]^ The resistance sensor area consisted of Mg traces 10 µm thick, with a resistance of 29 KΩ at room temperature, 130 times higher than the wider wavy‐shaped connectors. The sensor was covered by Si_3_N_4_ and SiO_2_ dielectric layers and encapsulated by Ecoflex. Results demonstrated that the sensor exhibited a linear dynamic response across a broad temperature range, with no hysteresis and a response time of 10 ms. With appropriate encapsulation, the sensor achieved stable operation for up to one day. The sensor completely dissolved after 67 days in a water–NaCl solution (150 m mol). Aside from metals, fibers exhibit excellent temperature response characteristics, making them suitable for use in temperature sensors. A flexible temperature sensor based on carbonized silk nanofibers was fabricated to measure the temperature of the extracellular environment.^[^ ^135^ ^]^ The temperature sensor had a high sensitivity (1.75%/°C) and a wide operating range (35–63 °C), as well as robust detection under bending situations. The sensor also showed good degradation; it degraded after ≈20 s under a magnetic stirrer when it was put into 0.1 m NaOH solution. In addition, the degradation rates of the substrate and the encapsulation layer were also investigated due to their greatly affecting the degradation rate of the device. The SF/PU composite film degraded rapidly when it was dropped in 0.1 mol L^−1^ NaOH solution. This ultrafast degradation ability greatly reduced the environmental decomposition burden and decomposition cycle, which fully proved that the sensor based on CSNF‐Pt was environmentally friendly. Han et al. developed the super‐elastic and biodegradable poly(l‐lactide‐co‐ε‐caprolactone) (PLCL) elastomer.^[^ ^91^ ^]^ As shown in **Figure** **5**a, PLCL was utilized in smart soft robotic grippers that could perceive environmental stimuli through integrated temperature sensors. The temperature‐sensing system enabled the gripper to respond to temperature changes below 80 °C. When approaching the threshold, the gripper was able to release the high‐temperature object on time, demonstrating the innovative potential of PLCL in soft robotics and transient electronics.

In addition to temperature, polymer‐based sensors offer advantages in humidity detection. A biodegradable hygroscopic soft robot for visual humidity sensing was developed by Mariani et al.^[^ ^136^ ^]^ The robot was based on degradable polymers, including poly(ε‐caprolactone) (PCL), polyethylene oxide (PEO), and cellulose nanocrystals (CNC). The structure was based on a biomimetic bilayer structure inspired by the seed of Pelargonium appendiculatum. What's more, the environmental humidity caused asymmetric expansion and significant structural deformation in the robot, enabling flexible bending, as shown in Figure 5b. Autonomous, battery‐free humidity sensing was enabled, and real‐time humidity levels could be visually detected with high sensitivity and accuracy. A wide detection range of 30%–90% RH, a resolution of 0.17%–0.52% RH, and an accuracy up to 98% were achieved. Another high‐performance humidity sensor made entirely of biodegradable biomaterials was reported by Wang et al.^[^ ^137^ ^]^ The electrodes of the sensor were fabricated by evaporating Mg/Fe (60/10 nm thick) on top of a 20 µm thick functionalized polysaccharides biocomposite film via spin coating and thermal evaporation. These devices exhibited high‐humidity sensitivity, great flexibility, and excellent noncontact characteristics. In addition, the device could completely decompose after its service life. The sensor dissolved by a relatively uniform hydrolysis process. The Fe/Mg electrodes degraded quickly at pH 5.5 at room temperature, usually within 25 min. The device degraded completely within 35 min.

Temperature and humidity are the two most commonly detected factors by sensors. In addition, depending on the specific application scenarios, sensors are designed to monitor other environmental conditions in order to achieve desired functionalities. Reprogrammable, recyclable origami robots controlled by magnetic fields were developed by Chung et al.^[^ ^138^ ^]^ They utilized water‐soluble tape (composed of wood fiber and carboxymethyl cellulose) as matrix material and mixed it with neodymium MPs to produce magnetic paper. The origami robot integrated with a photodiode could locate a desired place by modulating an external magnetic field and measuring photoinduced voltage changes relative to the surrounding light intensity, as shown in Figure 5c. Nasseri et al. developed programmable nanocomposites of cellulose nanocrystals and zwitterionic hydrogels for soft robotics.^[^ ^139^ ^]^ The novel hydrogel was applied in a pH‐responsive soft microgripper. When the pH value increased, the gripper was triggered to capture light spherical cargo by rotating the arm. After transferring the object, the pH‐reducing value enabled the gripper arm to open, allowing the release of the object. Furthermore, the safety for biomedical applications requiring biodegradability was supported by the on‐demand degradation capability of the material in saline solution, as shown in Figure 5d.

Building on this, multimodal sensors are developed to enable multidimensional perception of the environment.^[^ ^140^, ^141^ ^]^ For example, Alexandre et al. reported humidity and temperature sensors by aerosol jet printing and cellulose‐based substrates.^[^ ^142^ ^]^ The sensor used a biocompatible conducting polymer PEDOT:PSS, to create two distinct sensor types: a humidity sensor and a temperature sensor. This humidity sensor achieved high sensitivity (12.16% RH^−1^ in the range of 10%–80% RH) and demonstrated potential for real‐time health monitoring through human respiration tracking. Additionally, by crosslinking PEDOT:PSS with (3 Glycidyloxypropyl)Trimethoxysilane (GOPS), the sensors effectively detected changes within a temperature range of 20–50 °C. OH et al. proposed a lifetime‐configurable soft robot.^[^ ^133^ ^]^ The material used for the soft robot exhibited excellent mechanical stretchability and could degrade under ultraviolet light by mixing a fluoride‐generating diphenyliodonium hexafluorophosphate with a silicone resin. Importantly, flexible environmental sensors, including strain, temperature, and UV sensors, were embedded to endow the robots with multifunctionality, such as environmental monitoring and intelligent self‐destruction, as shown in Figure 5e.

#### 3.2.3 Olfactory Sensor

In industrial, agricultural, and aerospace production activities, toxic and hazardous gases such as hydrogen sulfide, chloride compounds, ammonia, and nitrogen oxides are often released or leaked. When such gas leakages occur, it is crucial to promptly trace the origin of the leakages. However, sending human workers to conduct source tracing and maintenance often poses significant life‐threatening risks. Embodied intelligent machines can effectively address this challenge. But in confined spaces, neither humanoid robots nor robotic dogs can perform adequately. In contrast, soft‐bodied robots can access narrow and complex environments to perform gas leakage tracing and even carry out the repairments due to their inherent flexibility and unique actuation mechanisms.

**Figure 6:**
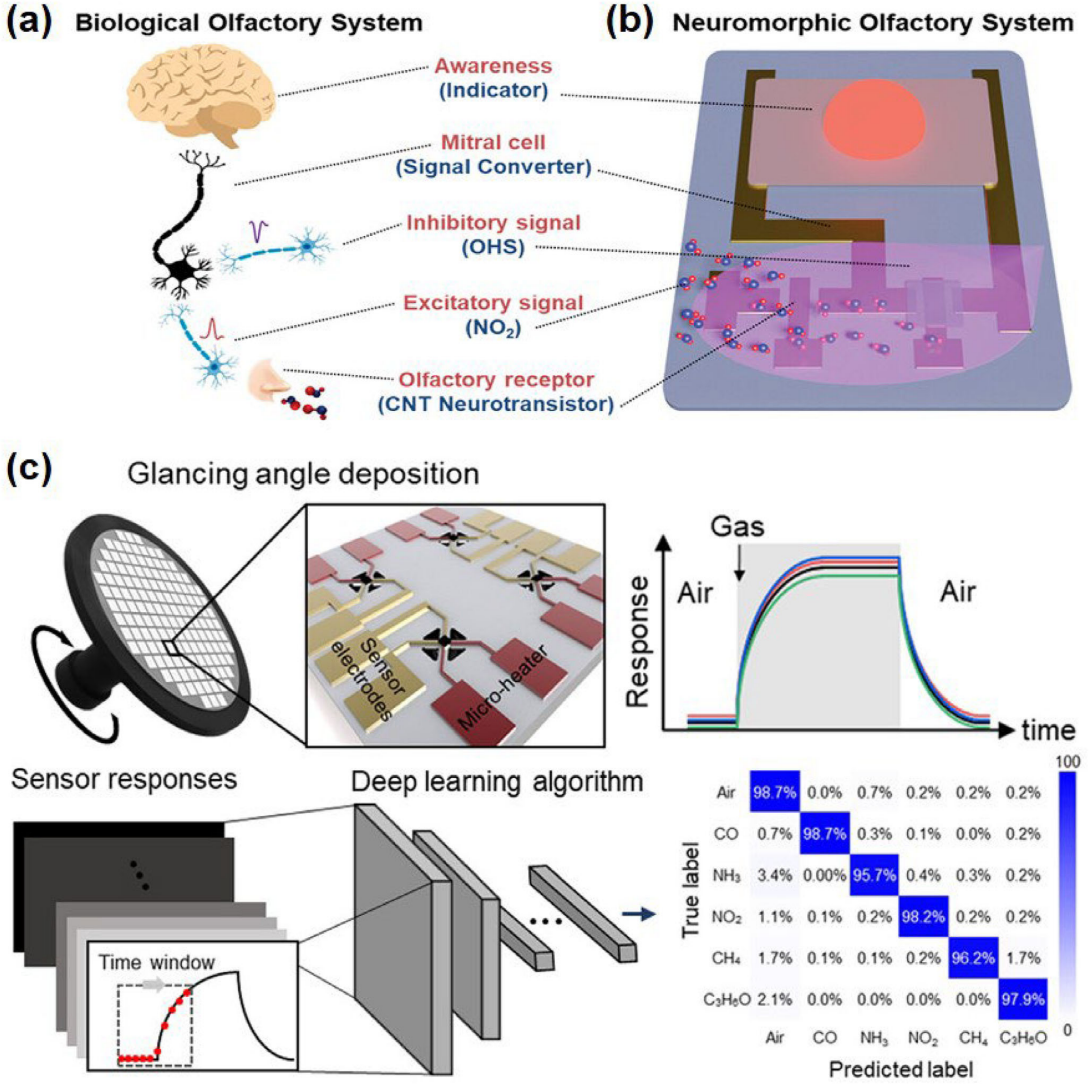
Comparative overview of the human biological a) and neuromorphic b) olfactory system. Reproduced with permission.^[^ ^143^ ^]^ Copyright 2024, John Wiley & Sons. c) Illustration of sensor array fabrication and gas classification using a convolutional neural network. Reproduced with permission.^[^ ^144^ ^]^ Copyright 2022, American Chemical Society.

To endow soft robots with this capability, they must be equipped with artificial olfactory systems. The nose, as one of the five major human sensory organs, performs olfactory functions through the following physiological process: gas molecules bind to receptor proteins on the surface of olfactory receptor cells located in the olfactory mucosa at the top of the nasal cavity. This binding triggers neural impulses in the receptor cells, which are then transmitted to the olfactory bulb in the brain. Subsequently, the olfactory bulb relays these signals to the central olfactory nervous system for further processing and analysis, enabling humans to perceive and distinguish various odors,^[^ ^143^ ^]^ as illustrated in **Figure** **6**a. An artificial olfactory system is a system designed to mimic this human olfactory functionality, with its schematic representation shown in Figure 6b. The neuromorphic olfactory system consisted of a neuro transistor, a load transistor, and a gas indicator. The system could exhibit analogous gas synaptic signal responses to gas exposure, providing the relevant functionality of its biological counterpart. Since the selectivity of a single gas sensor is normally not good, a sensor array is proposed to detect the target gases. As shown in Figure 6c, Park et al.^[^ ^144^ ^]^ proposed to construct a sensor array through the glancing angle deposition method and utilized the deep learning algorithm to identify the gas types and quantify the gas concentration. The convolutional neural network (CNN) using the input data in a matrix form was adopted as a learning algorithm, which could conduct pattern recognition of the sensor responses. Finally, real‐time selective gas detection for CO, NH_3_, NO_2_, CH_4_, and acetone (C_3_H_6_O) gas was achieved with an accuracy of 98% by applying preprocessed response data to the CNN.

**Figure 7:**
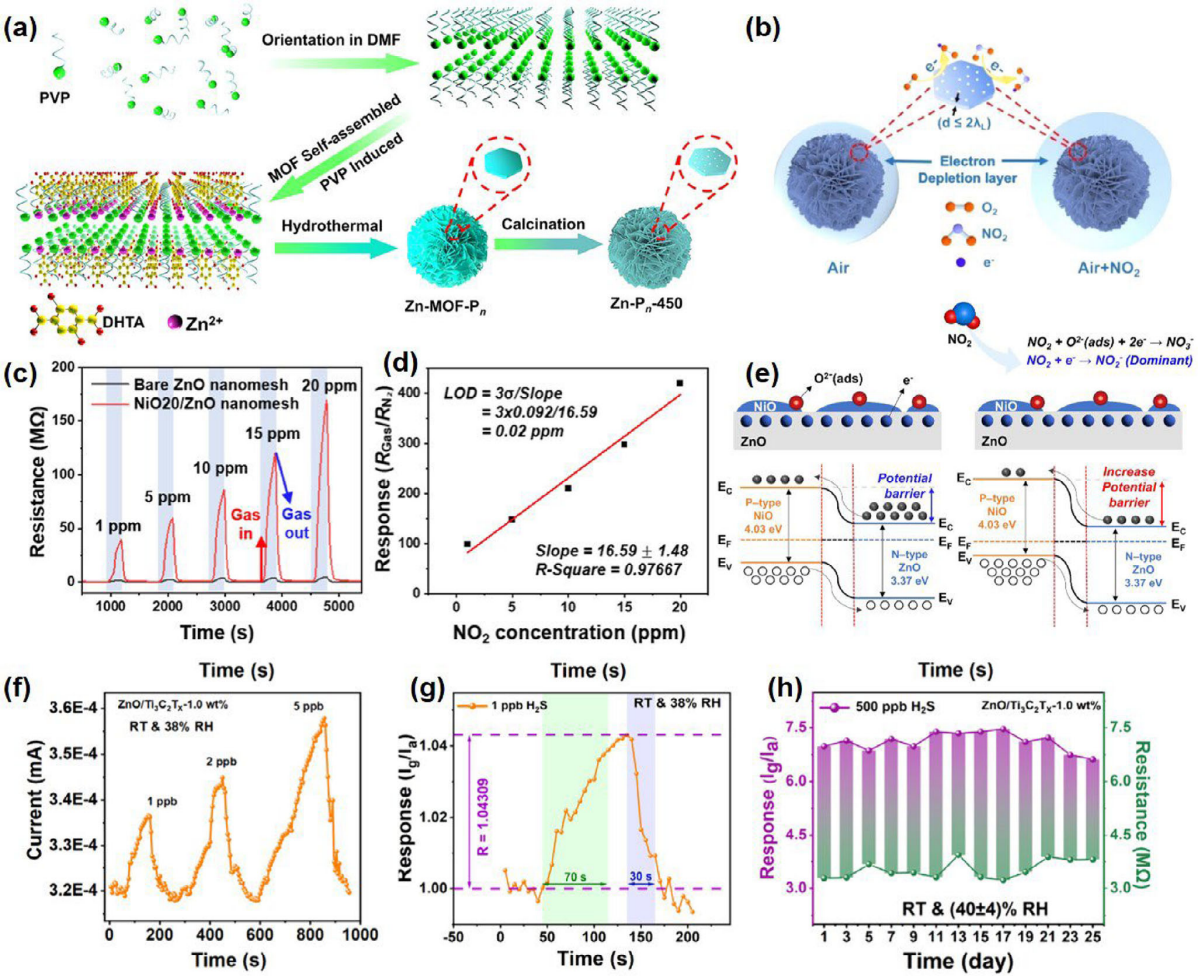
a) Schematic diagram of 2D porous ZnO synthesized through pyrolysis of the Zn‐MOF. Reproduced with permission.^[^ ^146^ ^]^ Copyright 2024, American Chemical Society; b) Mechanism model of NO_2_ gas sensing process^[^ ^146^ ^]^ Copyright 2024, American Chemical Society; c) Gas sensing performances of the NiO/ZnO nanomesh and d) the plots of responses as a function of NO_2_ concentration. e) Proposed sensing mechanism of the NiO/ZnO nanomesh: energy band diagram of the *p*‐type NiO/n‐type ZnO heterostructure and its gas‐sensing mechanism in N_2_ and NO_2_ environments. Reproduced with permission.^[^ ^147^ ^]^ Copyright 2025, American Chemical Society; f) real‐time sensing current to low concentrations (1–5 ppb) of H_2_S, g) dynamic response curve to 1 ppb H_2_S, and h) stability test. Reproduced with permission.^[^ ^148^ ^]^ Copyright 2025, American Chemical Society.

As a typical biodegradable material, ZnO has been widely utilized for various hazardous gas detection. ZnO is a degradable metal that generally remains stable under normal conditions. But in certain environments, particularly under acidic or highly humid conditions, ZnO may be gradually dissolved. A novel degradable aerogel based on lignin containing cellulose nanofibers and ZnO was innovatively prepared for ammonia detection at room temperature.^[^ ^145^ ^]^ The composite aerogel was fabricated through the simple processes involving hydrothermal reaction and freeze–drying. The incorporation of a small amount of carbon nanotubes significantly enhanced the sensing performance. At a room temperature concentration of 50 ppm ammonia, the aerogel demonstrated a response value of 4.94%. Furthermore, the aerogel sensor exhibited good water‐soluble degradability and soil biodegradability. In a water bath via ultrasonication, the degradable aerogel dispersed within 165 s. After being buried in grassland soil for 28 days, the degradable aerogel had almost completely degraded. Guo et al.^[^ ^146^ ^]^ reported a 2D porous ZnO‐based NO_2_ gas sensor, which was synthesized through pyrolysis of the Zn‐MOF as shown in **Figure** **7**a. Owing to its large surface area, abundant active sites, and rapid charge transfer, the 2D porous ZnO exhibits excellent performance both in sensitivity and selectivity, and the optimal sample could achieve a response value of 162 at the working temperature of 160 °C, which is 10 times higher than that of pristine ZnO. The gas sensing mechanism of the ZnO sensor was proposed and is illustrated in Figure 7b. When the *n*‐type ZnO is placed in an air atmosphere, the O_2_ molecules would adsorb on the surface of ZnO, and the attached O_2_ molecules captured the electrons from the conduction band, and turn into the adsorbed oxygen ion, where the species of oxygen anions depended on the operating temperature. Meanwhile, the energy band curvature occurred in the ZnO sensor, and the electron‐depleted layer formed on the ZnO surface. When the ZnO sensor was under the NO_2_ atmosphere, some NO_2_ molecules would adsorb on ZnO instead of O_2_, and the electrons of ZnO transferred to NO_2_ molecules, while other NO_2_ molecules reacted with the oxygen ion to form nitrite. Due to more electron transfer from ZnO to the NO_2_ molecule, a broader electron depletion layer was produced on the surface of the sensor. A larger band curvature occurred, along with the higher surface barrier obtained in the NO_2_ than in the air. To address the poor sensitivity of the ZnO, Cho et al.^[^ ^147^ ^]^ utilized an electrospun template‐assisted nanostructuring approach to form NiO/ZnO heterostructure, which is highly sensitive to the NO_2_ gas (Figure 7c). Through this strategy, significantly enhanced nitrogen dioxide sensitivity at 300 °C (*R*_Gas/_ *R*_N2 _= 420 at 20 ppm), along with rapid response (200 s) and recovery (50 s) times, and a detection limit as low as 0.02 ppm were observed. As shown in Figure 7d, good linearity is observed between the response and the gas concentration. Figure 7e presents the energy band diagram of the NiO/ZnO and its gas‐sensing mechanism. What is more, ZnO is an excellent sensitive material for H_2_S gas detection. Cui et al.^[^ ^148^ ^]^ constructed ZnO/Ti_3_C_2_T_X_ nanocomposite by combining ZnO nanoparticles, prepared by a hydrothermal method, with Ti_3_C_2_T_X_ MXene for H_2_S detection. Figure 7f shows that the ZnO/Ti_3_C_2_T_X_ is capable to detect 1 ppb H_2_S, with the response and recovery times of 70 and 30 s (Figure 7g), respectively. As shown in Figure 7h, after being placed for a month, the sensor still exhibited a response of 6.98(purple), with only 5% loss, while the initial resistance (green) remained stable near 3.6 MΩ.

#### 3.2.4 Biomedical Sensor and Implantable Sensor

An important category and development direction of soft robots is medical robots, such as drug delivery robots. Accurate detection of key biomarkers is crucial for medical robots, as it provides essential capabilities for real‐time monitoring, early diagnosis, and therapeutic activities. Therefore, developing biomedical sensors and implantable sensors for medical robots to detect biomarkers is an important research endeavor. These biomarkers include various ions (e.g., K^+^, Na^+^, Ca^2+^), small molecules (e.g., H_2_O_2_, lactic acid, glucose, O_2_, NO), and large organic molecules such as mRNA. However, if the sensors are non‐degradable, a high‐risk secondary surgery is required to remove them. Biodegradable implantable sensors are able to naturally degrade through biological processes while offering all the advantages of traditional implantable sensors. Their self‐degradation properties eliminate the need for surgical removal, thereby reducing patient discomfort and tissue damage.

The concentration of H^+^ ions in the body, that is, the pH value, is crucial for acid‐base balance regulation. Monitoring the pH value within the body can help identify or predict various diseases, such as predicting cancer growth and heart disease. Barillaro et al. reported a bioresorbable nanostructured pH sensor, which consisted of a nanostructured porous silica scaffold conformably coated with a nanometer‐thick multilayer of fluorescent polymers.^[^ ^149^ ^]^ Due to the biodegradability of porous silica, fluorescent polymer, and the PLGA substrate, the pH sensor would be fully degraded in one week after implant body. By using polyelectrolytes with a lower degradation rate, the lifespan of sensors could be extended and adjusted according to specific needs.

The Ca^2+^, K^+^, and Na^+^ ions in the cerebral cortex can synergistically reflect the neurophysiological states. Yang et al. have fabricated a flexible multifunctional electrode (FME) based on a carbon nanotube array to record electrocorticography and extracellular ions of Ca^2+^, K^+^, and Na^+^ on the surface of the cerebral cortex.^[^ ^150^ ^]^ The FME was carefully attached to the surface of the rat's cerebral cortex and achieved effective monitoring of electrophysiology and detection of Ca^2+^, K^+^, and Na^+^ on the cerebral cortex for 2 weeks.

Cardiovascular and cerebrovascular diseases, with their high mortality and disability rates, make timely treatment critical for saving lives and improving clinical outcomes. Early detection and timely treatment through scientific monitoring methods are key measures in preventing condition deterioration and reducing mortality risks. Wang et al. fabricated a biodegradable and restorative neural interface designed for concurrent monitoring and facilitating the healing of long‐gap nerve injuries at a finite time frame matched to the critical initial phase of nerve recovery.^[^ ^151^ ^]^ This device consisted of a 7‐channel electrode array for signal recording and a biodegradable galvanic cell to promote nerve regrowth. The electrode array was a bilayer structure comprising Mo thin films (300 nm) and highly doped N‐type monocrystalline Si membranes. The electrodes were deposited on a flexible, biodegradable substrate. A biodegradable galvanic cell, composed of thin‐film magnesium (Mg, 3.5 µm) and iron‐manganese alloy (FeMn, 1.5 µm) electrodes, was integrated next to the Mo/Si array. The novel cell could provide electrical stimulation for nerve regeneration. The device also included a biodegradable shape memory polymer, which enables self‐rolling around peripheral nerves at physiological temperatures (37 °C), facilitating implantation. The device exhibited controlled degradation in phosphate‐buffered saline solutions at 60 °C. The Mo contacts coated with biodegradable polymer dissolved in ≈9 days, while Si degraded more slowly, estimating a 5.5–8.5 week lifespan. Polymeric substrates are fully hydrolyzed within ≈20 days.

**Figure 8:**
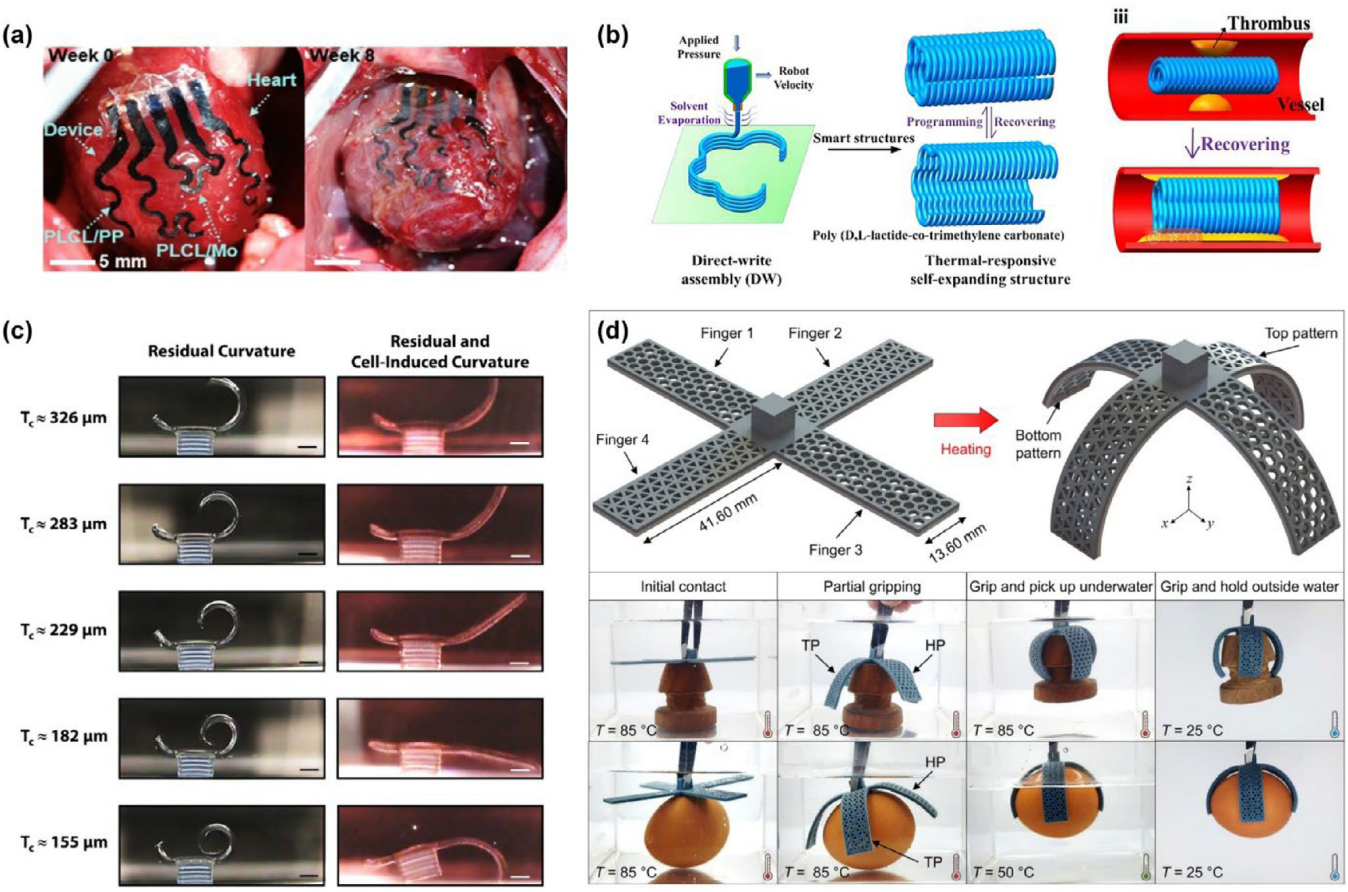
The application of biodegradable devices in the biomedical field. a) Flexible sensors based on degradable materials used for heart protection. Reproduced with permission.^[^ ^91^ ^]^ Copyright 2023, Springer Nature. b) Biodegradable PLMC material printed into vascular stents by 4D printing. Reproduced with permission.^[^ ^153^ ^]^ Copyright 2019, John Wiley & Sons. c) The actuating process of bio‐bots through residual and cell‐induced surface stresses. Reproduced with permission.^[^ ^154^ ^]^ Copyright 2012, Springer Nature. d) The working principle of a polylactic acid‐based actuator and the process of grasping objects. Reproduced with permission.^[^ ^155^ ^]^ Copyright 2022, MDPI.

Post‐cardiac surgery monitoring is crucial for maintaining health and detecting postoperative complications early. To avoid the risks associated with a second complex surgery to remove cardiac sensors, a biodegradable and multifunctional integrated sensor has been developed.^[^ ^152^ ^]^ The sensor array was meticulously crafted using biocompatible and biodegradable materials, including a substrate of biodegradable PLA and a biodegradable Mg metallic electrode as the primary conductive element. Then, the array was further enhanced with the incorporation of Zn nanoparticles, which were utilized to form the functional sensing layers of the various sensors. The device features two electrochemical biosensors for detecting lactate and pH levels, a pressure sensor, and a chemical resistor array for detecting volatile organic compounds. The degradation of the Mg electrodes was demonstrated and proven in the simulated body fluid (SBF), which completely degraded within 24 h. The Zn NPs degraded within 2 months. The degradation of the PLA took longer, taking ≈1 year to dissolve completely in the SBF. Additionally, another ultra‐stretchable and biodegradable elastomer that could be used for implantable cardiac sensors was fabricated by Han et al.^[^ ^91^ ^]^ The elastomer could be stretched up to ≈1600%, demonstrating excellent properties in toughness, tear‐tolerance, and storage stability, as shown in **Figure** **8**a. The biodegradable elastomers could be applied in soft robotic grippers and cardiac implants. Breakthrough innovations in soft robotic grippers and cardiac implantable sensors enabled the development of adaptive bioelectronic interfaces for robotic‐assisted surgery and closed‐loop cardiovascular therapeutics. This integrated platform demonstrated multi‐scale compatibility via systematic verification processes. The experimental validation confirmed its profound integration capacity with next‐generation biomedical cyber‐physical systems.

Besides implantable sensors, biodegradable materials also play an important role in the field of minimally invasive biomedicine. Wan et al.^[^ ^153^ ^]^ engineered biocompatible poly (D,L‐lactide‐co‐trimethylene carbonate) (PLMC) actuator via direct ink writing additive manufacturing, as shown in Figure 8b. The 4D‐printed devices exhibited spatiotemporally programmable shape transformations under thermal stimulation. The printed PLMC actuator demonstrated its potential as a 1D surgical suture line, 2D non‐woven fabric, and 3D self‐expanding stent, which could be used in minimally invasive biomedical applications.

In the field of biomedicine, in addition to implantable medical devices, in vivo drug delivery is also an important therapeutic approach. Miniature flexible robots based on biodegradable materials and human cells can achieve drug transportation within the human body, thus holding great potential for widespread application. Chan et al.^[^ ^154^ ^]^ fabricated a “biological robot” consisting of hydrogel and cardiac cells via 3D printing, as shown in Figure 8c. The multi‐material biological robot was composed of a “biological bimorph” cantilever structure and a base structure: the former achieved power‐driven motion by inoculating a layer of contractile cardiac cells, while the latter regulated the direction of movement through shape design. The biological robot had great potential in the fields of biosensing, drug delivery, energy production, environmental restoration, and artificial immune systems.

Compared with micro‐robots introduced into the human body, flexible robots can grasp and transport objects flexibly. Additionally, biodegradable materials enhance the application potential of flexible robots in fields such as healthcare and logistics. Alshebly et al.^[^ ^155^ ^]^ developed a thermally actuated hand‐like gripper using geometrically patterned biodegradable polylactic acid (PLA), as shown in Figure 8d. This biomimetic device demonstrated adaptive grasping capabilities for both uniform and non‐uniform objects, exhibiting potential for deployable self‐morphing architectures and soft robotic systems.

Biodegradable materials hold great potential for medical applications, particularly in implantable biosensors, but selecting those that meet both performance and regulatory standards presents significant challenges. Materials must demonstrate the required mechanical strength, electrical performance, and biodegradability, while also complying with medical‐grade standards such as ISO 10993 and USP Class VI to ensure biocompatibility and safety. The regulatory approval process is complex, requiring thorough evaluation of biodegradation rates, chemical stability, and potential toxicity. Balancing the performance of materials in a biological environment with their degradation without adverse effects is a critical challenge. This requires careful consideration in medical device development.

## 4 Challenges

Although significant progress has been made in soft robots, their development still faces critical challenges requiring breakthroughs ranging from materials science, sensing systems, energy technology, and manufacturing processes.

First, in terms of materials, it is challenging to improve flexibility and biodegradability while retaining electrical performance without reducing strength or hastening deterioration. The use of biodegradable materials may come at the cost of reduced sensor sensitivity. Therefore, designing soft robots necessitates a balance between material properties and sensing capabilities. Specifically, soft robots require materials that offer excellent flexibility and stretchability to meet the demands of complex motion, while simultaneously requiring superior sensing performance to ensure precise environmental perception. However, these properties often involve trade‐offs: overly flexible materials may compromise sensing performance, while overly rigid materials may limit adaptability. Thus, developing materials that integrate both mechanical robustness and sensing functionality is one of the key research directions. Future solutions may involve designing multifunctional composite materials or incorporating novel nanomaterials to achieve this balance.

Developing biodegradable materials for actuators remains a challenge. Traditional actuator materials, such as shape memory alloys, are typically incompatible with biodegradable materials. Novel biodegradable materials, such as biological hydrogels suitable for actuators, still need further development. The issue is also significant for the fabrication of fully biodegradable soft robots. Currently, some studies have achieved the goal of full degradation through the use of biodegradable materials. For example, Oh et al. reported an on‐demand transient and hyperelastic robotic material for lifetime configurable soft robots.^[^ ^133^ ^]^ This material was made by dispensing a photoinduced fluoride‐generating diphenyliodonium hexafluorophosphate (DPI‐HFP) to various silicone resins (Sylgard‐184 and Ecoflex 00–30), which will be complete decomposition after processing by UV light (365 nm, 30 min) and followed by heat treatment (120 °C, 60 min). However, fully biodegradable soft robots are still relatively scarce at present. Developing and expanding a wider variety of biodegradable materials will help unlock their potential and drive further progress in this field.

What's more, ensuring the long‐term stability of biodegradable materials is also a significant challenge. The degradation process often results in unpredictable changes in mechanical properties, which can significantly affect the performance and reliability of the system over time. In particular, developing biodegradable materials for actuators presents a unique difficulty. Traditional actuator materials, such as shape memory alloys, are typically incompatible with biodegradable materials. While novel biodegradable materials like biological hydrogels show promise for actuators, further development is required to ensure they offer both functional stability and long‐term durability.

Additionally, the adaptability of soft robots in dynamic and uncertain environments requires further enhancement. For instance, in industrial settings, they need to respond rapidly and adjust to highly dynamic production lines. In contrast, in medical applications, these robots are required to navigate complex biological environments while performing tasks with a high degree of accuracy. Moreover, in the medical field, achieving a balance between performance and regulatory compliance presents significant challenges. Ensuring that soft robots meet both functional requirements and medical‐grade standards, such as biocompatibility and safety, adds complexity to their design and implementation. Current limitations in environmental perception and adaptability highlight the need for improved sensor technologies, advanced AI algorithms, and more flexible control strategies. For example, integrating deep learning with real‐time sensing could enable soft robots to better recognize and respond to environmental uncertainties.

Another major challenge lies in the large‐scale, low‐cost production of biodegradable soft robots. Many manufacturing processes, such as 3D printing or micro‐nano fabrication, are complex and hinder mass production. Furthermore, advanced materials in flexible electronics often incur high costs. Addressing these issues requires developing cost‐effective, high‐efficiency manufacturing techniques. Future advancements may involve optimizing production workflows, adopting automated manufacturing technologies, or exploring new low‐cost biodegradable materials to reduce overall expenses.

## 5 Future Perspectives

The development of soft robots is advancing toward intelligence, multifunctional integration, and green sustainability. By integrating artificial intelligence (AI) and self‐learning algorithms, soft robots will achieve enhanced environmental adaptability and task execution capabilities. For instance, machine learning algorithms can train robots to autonomously navigate complex environments, identify obstacles, and optimize path planning. A notable example is intelligent soft medical robots, which leverage self‐learning algorithms to enable real‐time perception of patient anatomy and surgical path optimization for precision procedures. In disaster response, autonomous soft robots can conduct search and rescue operations in hazardous environments, minimizing risks to human responders.

Multifunctional integration represents another critical direction for soft robots. Advanced sensing modules—equipped with tactile sensors for detecting physical contact, visual sensors for environmental mapping, and force sensors for measuring interaction dynamics—allow robots to dynamically respond to environmental changes. Green and sustainable development is also a key focus. Material selection and production processes increasingly prioritize eco‐friendly solutions, such as recyclable and biodegradable materials, to reduce environmental impact. Recyclable materials enable end‐of‐life robot components to be repurposed, curbing resource waste and pollution. Biodegradable materials, meanwhile, allow robots to decompose naturally post‐mission, avoiding long‐term ecological harm. Sustainability efforts also extend to minimizing energy consumption and carbon emissions during manufacturing. By optimizing production techniques and adopting renewable energy sources, the lifecycle of soft robots will align more closely with environmental stewardship. The use of self‐healing materials is also a strategy to extend the lifespan and improve the reliability of soft robots.

## 6 Conclusion

Degradable materials have gradually become a popular choice in the design of sustainable soft robotics due to their excellent environmental adaptability and degradation performance. The use of degradable materials brings numerous advantages to soft robotic design. First, these materials significantly reduce environmental pollution caused by soft robots, particularly in medical and environmental monitoring fields, where their biodegradable properties can avoid ecological issues associated with the long‐term residue of traditional soft robots. Second, the flexibility and biocompatibility of biodegradable materials enable them to better adapt to complex application scenarios. Additionally, these materials offer new possibilities for functional integration in soft robots, such as achieving self‐destruct or recycling of soft robots through their degradation properties, thereby further enhancing the system's intelligence level. However, the development of sustainable soft robots based on biodegradable materials remains at an early stage, and a completely biodegradable soft robotic system has yet to be realized. One reason is that soft robotics consists of multiple components, such as actuators and sensors, which are made from various materials. Challenges arise in balancing the degradation performance of materials with the functionality and stability of actuators and sensors, optimizing material synthesis processes to reduce costs, and expanding their applications in complex environments. Future research must foster closer interdisciplinary collaboration between materials science, sensor technology, and soft robotic design to accelerate progress in this field. Biodegradable materials open up new possibilities for the design and application of soft robotic sensors.

## Conflict of Interest

The authors declare no conflict of interest.
